# The role of the cGAS-STING-mediated inflammatory amplification loop in osteoarthritis: from danger-associated DNA sensing to whole-joint remodeling and precision therapy

**DOI:** 10.3389/fcell.2026.1995231

**Published:** 2026-10-28

**Authors:** Xuxu Yang, Lin Zhang, Yang Yu, Shuhong Wu, Lidan Yang

**Affiliations:** Department of Orthopedics, Affiliated Hospital of Zunyi Medical University, Zunyi, China

**Keywords:** cGAS–STING, danger signals, inflammatory amplification, mitochondrial DNA, osteoarthritis, precision intervention

## Abstract

Osteoarthritis (OA) is a heterogeneous disorder of the entire joint in which mechanical overload, aging, metabolic disturbance, and dysregulated immunity converge. Growing evidence places the cyclic guanosine monophosphate–adenosine monophosphate synthase (cGAS)–stimulator of interferon genes (STING) pathway at the interface between cellular damage and sterile inflammation because this pathway detects self-deoxyribonucleic acid (DNA) that escapes its normal intracellular compartments. This review interprets current findings through a danger-signal amplification framework rather than treating cGAS–STING as an isolated inflammatory pathway. Mechanical and metabolic stress, oxidative injury, and aging can compromise mitochondrial and nuclear integrity; among the resulting DNA species, cytosolic mitochondrial DNA (mtDNA) currently has the strongest experimental support as an endogenous activator of cGAS in OA. cGAS-generated 2′3′-cyclic guanosine monophosphate–adenosine monophosphate (2′3′-cGAMP) activates STING and engages TANK-binding kinase 1 (TBK1)–interferon regulatory factor 3 (IRF3)/type I interferon (IFN-I) and nuclear factor kappa B (NF-κB) programs. These responses intersect with inflammasomes, pyroptosis, cellular senescence, and the senescence-associated secretory phenotype (SASP), thereby linking DNA exposure to renewed tissue injury. Extracellular vesicles (EVs), including vesicles carrying mtDNA, can extend this process across cartilage, synovium, the infrapatellar fat pad, and subchondral bone and help convert focal cellular stress into whole-joint remodeling. Potential interventions therefore span several levels: preventing mtDNA damage or escape, restoring mitochondrial quality control, directly modulating cGAS or STING, attenuating downstream inflammatory and cell-death programs, and improving tissue-selective local delivery. A major translational priority is to define when and in which OA endotypes cGAS–STING activity is sufficiently dominant to justify pathway-directed treatment. Combining temporal analysis, cell-specific models, and mechanism-linked biomarkers may ultimately support stage-adapted therapy that controls danger signals while preserving physiological innate immune function.

## Graphical Abstract

**Figure:**
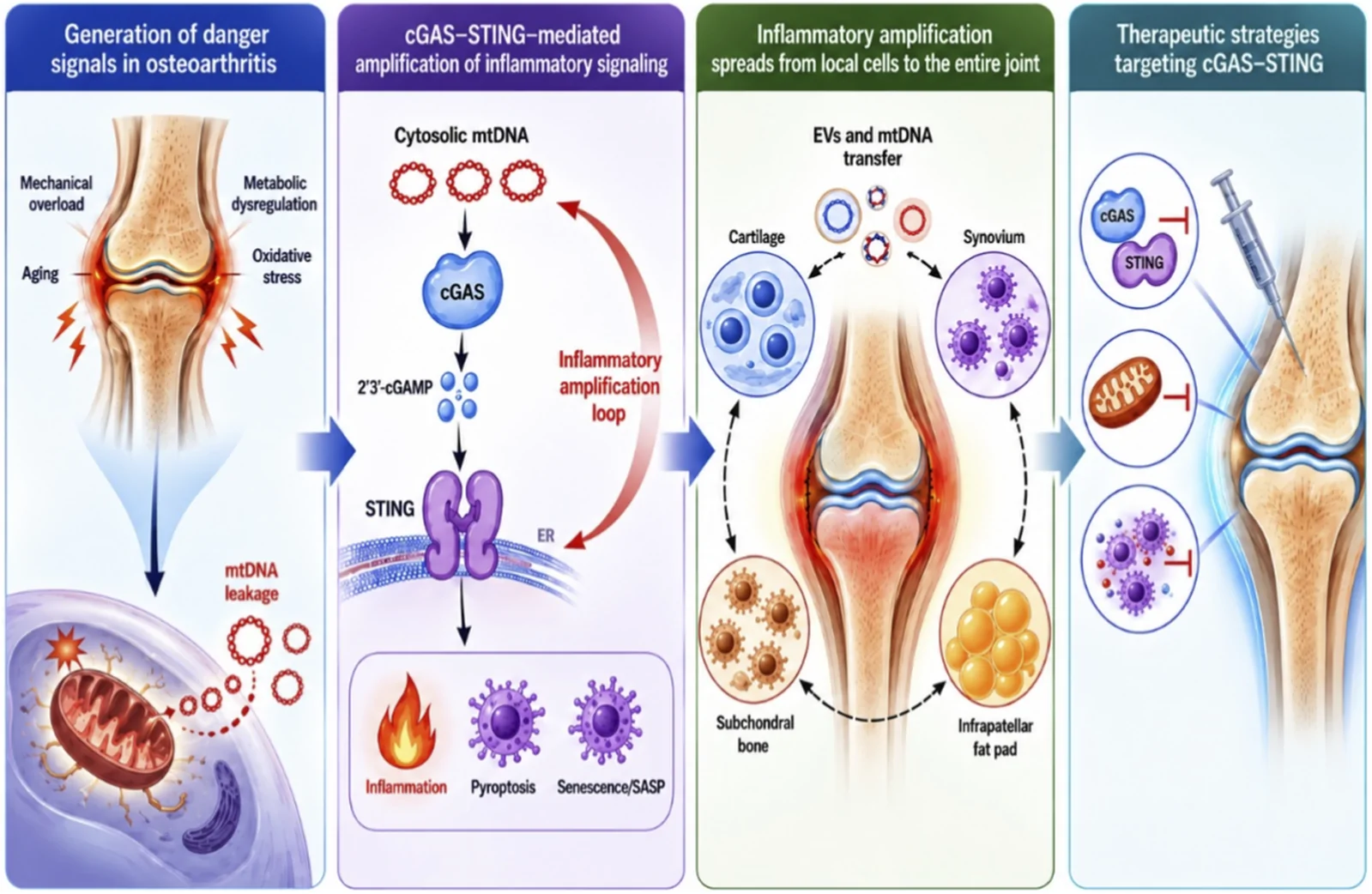

## 1 Introduction

Osteoarthritis (OA) is a chronic degenerative joint disease characterized primarily by articular cartilage degeneration and accompanied by synovial inflammation, subchondral bone remodeling, osteophyte formation, and changes in periarticular tissues (Ru et al., 2026). As understanding of its pathogenesis has advanced, OA is no longer viewed simply as localized cartilage injury caused by mechanical wear. It is increasingly regarded as a whole-joint disease driven by the combined effects of mechanical stress, aging, metabolic abnormalities, and immune dysregulation. Of particular importance, low-grade but persistent inflammation is present across multiple stages of OA. Interactions among synovial cells, macrophages, and injured chondrocytes can collectively shape the intra-articular inflammatory microenvironment and further influence cartilage destruction and disease progression (Sanchez-Lopez et al., 2022; Robinson et al., 2016).

The central question in OA inflammation is not merely whether inflammatory mediators are elevated, but why an initially limited mechanical or metabolic insult can gradually become a chronic, self-sustaining inflammatory state. Under normal conditions, danger signals generated by tissue injury should subside as damaged components are cleared and tissue repair proceeds. In OA joints, this balance may remain persistently disrupted. Mechanical overload, oxidative stress, mitochondrial dysfunction, and cellular senescence can damage cells and organelles and promote the generation of DAMPs (Lambert et al., 2020; Fernández-Moreno et al., 2022; Diekman and Loeser, 2024). Inflammation itself can then further accelerate matrix degradation, cellular senescence, and cell death, leading to continued release of new danger signals. The original injury therefore becomes more than the starting point of disease. Through innate immune sensing, it can be converted into renewed inflammatory stimulation, creating a pathological feedback process in which tissue damage promotes inflammation and inflammation generates further tissue damage.

Among the many DAMPs, mislocalized self-DNA has received particular attention in recent years. When nuclear or mitochondrial integrity is disrupted, nuclear DNA or mtDNA can become abnormally exposed in the cytosol, where cGAS functions as a major cytosolic DNA sensor. After binding cytosolic double-stranded DNA, cGAS synthesizes the second messenger 2′3′-cGAMP, which activates STING and engages downstream TBK1-IRF3 and NF-κB inflammatory programs (Yu and Liu, 2021; Kato et al., 2017). The cGAS-STING pathway is an important component of host innate defense against pathogens. However, when it continuously senses DNA released from damaged host cells, this defense system may become a major driver of sterile inflammation (Hopfner and Hornung, 2020). Dysregulation of cGAS-STING has been implicated in multiple inflammatory disorders associated with DNA damage and is increasingly recognized as an important immune-sensing pathway in OA.

Experimental studies have begun to provide direct evidence that cGAS-STING participates in OA progression (Zhou et al., 2025; Linde et al., 2025; Zhang et al., 2025; Chen X. et al., 2026). One study of human OA cartilage and experimental OA models found increased STING expression in OA cartilage. Enhanced STING activity promoted extracellular matrix (ECM) degradation, senescence, and apoptosis in chondrocytes, whereas STING knockdown attenuated these abnormalities and improved cartilage degeneration and synovial inflammation in experimental OA (Guo et al., 2021). More recently, research has moved beyond single-pathway activation toward explaining how inflammation becomes self-reinforcing in OA. A 2024 study identified a feed-forward loop in OA chondrocytes involving transient receptor potential melastatin 2 (TRPM2), calcium (Ca^2+^), mtDNA, cGAS, STING, and NF-κB. In this loop, mtDNA generated by mitochondrial injury promotes cGAS-STING inflammatory signaling, while NF-κB further enhances upstream TRPM2-related responses and thereby sustains chondrocyte damage (Sun K. et al., 2025). These findings suggest that the importance of cGAS-STING in OA extends beyond increasing the expression of selected inflammatory mediators. By linking cellular injury to innate immune responses, the pathway enables self-DNA generated by damage to be repeatedly converted into new inflammatory outputs.

Together, these findings point to a pathological pattern that warrants further attention. Mechanical, metabolic, and aging-related stress first damages the nucleus or mitochondria. Aberrantly released or accumulated self-DNA then triggers cGAS-STING signaling and promotes inflammation, cellular senescence, and cell-death programs. These changes further injure cells and the ECM, generating new sources of DAMPs and DNA. If this feedback process cannot be terminated by normal DNA clearance, organelle quality control, and inflammatory-resolution mechanisms, a local injury may gradually evolve into a persistent inflammatory amplification loop. At present, cGAS-STING can therefore be viewed as a context-dependent inflammatory amplification node whose causal contribution may vary with cell type, disease stage, nature of injury, and patient biological background.

Unlike previous reviews that have primarily summarized the components, activation mechanisms, and individual downstream effects of the cGAS-STING pathway, this review proposes a “danger-DNA amplification” framework. This framework places the generation and abnormal exposure of immunogenic DNA, cGAS-STING sensing, inflammatory and cell-fate feedback, EV-mediated intertissue propagation, and stage-specific therapeutic intervention within a single continuous pathological chain. It positions cGAS-STING as an important amplification node linking local tissue injury to a self-sustaining, intertissue inflammatory network across the whole joint. By further connecting this mechanistic framework with biomarkers, patient stratification, and precision intervention, the model may help clarify the actual role of cGAS-STING in chronic OA inflammation and provide a theoretical basis for identifying translational biomarkers and developing more precise targeted anti-inflammatory strategies.

## 2 Generation of danger signals in osteoarthritis

### 2.1 Mechanical loading and chondrocyte injury generate the earliest danger signals

Mechanical loading is essential for maintaining the normal biological function of articular cartilage. Within the physiological range, compression, tensile strain, and fluid shear regulate chondrocyte metabolism and maintain a dynamic balance between matrix synthesis and degradation. However, when the magnitude, duration, or distribution of loading becomes abnormal, the biological meaning of mechanical stimulation changes (Bachrach et al., 1995). Structural damage to the cartilage surface further concentrates local stress. When chondrocytes are chronically exposed to a mechanical environment that exceeds their adaptive capacity, the cytoskeleton, ion homeostasis, and organelle function can all be disrupted (Gratz et al., 2008; Huang X. et al., 2026). At this point, mechanical factors no longer act only as external forces that wear down the matrix. They also become upstream drivers of intracellular danger-signal generation.

Studies of the mechanosensitive ion channel Piezo1 have provided a more direct molecular explanation for this process. Sun et al. found that mechanical stress increased Piezo1 expression and Ca^2+^ influx in chondrocytes. Excessive Ca^2+^ increased mitochondrial Ca^2+^ loading and altered mitochondrial permeability, followed by release of mtDNA into the cytosol. Chondrocyte-specific deletion of Piezo1 reduced cytosolic DNA-associated immune responses and cartilage degeneration in experimental OA (Sun L. et al., 2025). Similar findings have been reported in temporomandibular joint OA. A 2025 study showed that abnormal mechanical stress on condylar chondrocytes induced the entry of mitochondria-derived double-stranded DNA into the cytosol and enhanced cGAS-STING-related responses (Dong et al., 2025). The observation of this phenomenon at different anatomical sites of OA suggests that stress-induced DNA mislocalization is not restricted to knee models. It may represent a more general stress response of chondrocytes to persistent mechanical overload.

Piezo1 is not the only route through which mechanical injury is transmitted. As the cartilage matrix degrades, tissue elastic modulus, osmotic pressure, and local stress distribution all change, exposing surviving chondrocytes to a physical microenvironment that differs substantially from healthy cartilage (Gru et al., 1989; Cooke et al., 2018). Studies have shown that Piezo1 can convert mechanical stress into chondrocyte senescence-related signaling. Abnormal Piezo1 activation induced by mechanical loading is also associated with impaired mitophagy and reduced mitochondrial function (Yu et al., 2025). As cartilage architecture deteriorates further, the same level of daily loading may therefore exert stronger damaging effects on the remaining cells, allowing early mechanical abnormalities to evolve into persistent intracellular stress. Mechanical and inflammatory factors should thus not be considered two independent pathogenic modules in OA. Abnormal loading first disrupts ionic and organelle homeostasis, allowing normally sequestered endogenous molecules to become danger signals. Loss of cartilage structure then alters subsequent loading conditions, making this process more likely to recur.

### 2.2 Mitochondrial dysfunction and mtDNA leakage constitute a major endogenous DNA source

Mitochondria have important roles in redox regulation, Ca^2+^ buffering, metabolic signaling, and cell-fate control in articular chondrocytes (Grishko et al., 2009). Mechanical overload, inflammatory stimulation, and oxidative stress in the OA environment increase mitochondrial burden, leading to loss of membrane potential, accumulation of reactive oxygen species, disturbed mitochondrial dynamics, and impaired clearance of damaged mitochondria (Xin et al., 2022). Once mitochondria shift from homeostatic regulators to damaged organelles, the consequence is not limited to reduced adenosine triphosphate (ATP) production. mtDNA, which is normally segregated from cytosolic immune-sensing systems, may also gain access to the cytosol.

mtDNA abnormalities in OA are not a recent observation. Grishko et al. reported that OA chondrocytes have more extensive mtDNA damage than normal chondrocytes and a reduced capacity to repair mtDNA lesions caused by oxidative and nitrative stress. After exposure to reactive oxygen species (ROS) or reactive nitrogen species (RNS), OA chondrocytes showed greater mtDNA damage and increased susceptibility to cell death (Grishko et al., 2009). These findings indicate that the mitochondrial genome in OA cells experiences greater oxidative pressure and has a reduced capacity to recover from injury. Whether mtDNA actually enters the cytosol determines whether mitochondrial damage can be converted into a danger signal recognizable by innate immunity. In 2026, Huang et al. showed that cytosolic mtDNA accumulation in OA chondrocytes is not simply an uncontrollable passive leak. They found that the ribonucleic acid (RNA)-binding protein tristetraprolin (TTP) limits cytosolic mtDNA release by regulating cytidine/uridine monophosphate kinase 2 (CMPK2), and that inhibition of CMPK2 markedly reduces mtDNA leakage (Huang H. et al., 2026).

The extent of mtDNA exposure is also determined by whether damaged mitochondria can be removed in a timely manner. Normal mitochondrial quality control limits the accumulation of dysfunctional organelles through mitochondrial fission, fusion, and selective autophagy. When this system cannot adequately process the continuous generation of damaged mitochondria, ROS production, abnormal membrane permeability, and release of mitochondrial contents are more likely to persist (Youle and van der Bliek, 2012; Li et al., 2023). Several recent OA studies have shown that restoring PTEN-induced kinase 1 (PINK1)/Parkin-related mitochondrial quality control can improve mitochondrial function and reduce cartilage injury. However, changes in PINK1/Parkin expression and mitophagic activity are not fully consistent across OA models, suggesting that mitophagy is influenced by disease stage and the nature of the stimulus and cannot simply be described as uniformly decreased or increased (Chen et al., 2025; Shin et al., 2019). This complexity does not diminish the importance of mitochondrial quality control in danger-signal generation. Rather, it suggests that the critical issue may not be the absolute abundance of a single autophagy protein, but whether the rate of damaged-mitochondria generation exceeds cellular clearance capacity. Once this balance is disrupted, oxidatively damaged mtDNA and mitochondria with abnormal permeability may accumulate together. Recent experimental interventions in OA further support this link. For example, enhancing mitophagy can reduce cGAS-STING-related inflammation and cell injury, suggesting that limiting the accumulation of damaged mitochondria may reduce the supply of immunogenic DNA at its source (Chen S. et al., 2026).

Compared with nuclear DNA, mtDNA currently has stronger evidence as an endogenous cGAS stimulus in OA. OA chondrocytes show clear mtDNA damage and repair defects, and mechanical stress, Ca^2+^ imbalance, and changes in specific regulators of mtDNA have been shown to affect cytosolic mtDNA levels. Mitochondria are therefore not only organelles affected by metabolic abnormalities in OA. They may also function as intracellular reservoirs that continuously generate danger DNA.

### 2.3 Nuclear DNA damage and aberrant DNA exposure

OA chondrocytes are chronically exposed to mechanical, oxidative, and inflammatory stress, which also compromises nuclear genome integrity. Early comparisons of normal and OA chondrocytes showed higher levels of genomic DNA damage in OA cells together with reduced coordination of gene expression (Copp et al., 2022). Subsequent work showed that cumulative DNA damage can induce abnormal expression heterogeneity resembling that of OA chondrocytes and linked this state to progressive stress-induced senescence (Rose et al., 2012).

The extent of DNA damage is determined not only by external stimuli but also by cellular repair capacity and chromatin homeostasis. Sirtuin 6 (SIRT6) is an important protein involved in DNA repair and telomere homeostasis. Reducing SIRT6 expression in human chondrocytes increases the DNA-break marker phosphorylated histone H2AX (γH2AX) and markers of telomere dysfunction, while also promoting cellular senescence and matrix metalloproteinase expression (Nagai et al., 2015). These findings indicate that as chondrocytes age and DNA-maintenance mechanisms decline, the same degree of oxidative or mechanical stress may leave more unrepaired nuclear DNA damage.

How nuclear DNA damage becomes accessible to cGAS requires more cautious interpretation. In other disease and cellular models, chromosome breakage or missegregation can generate micronuclei. When the micronuclear envelope ruptures, the chromatin inside loses effective separation from the cytosol, allowing cGAS to contact mislocalized genomic DNA (Harding et al., 2017). The landmark study by Mackenzie et al. showed that micronuclei arising from genomic instability can recruit cGAS, thereby establishing a mechanistic link between nuclear DNA damage and cytosolic DNA sensing (Mackenzie et al., 2017). Although DNA damage, senescence, and STING abnormalities in OA chondrocytes are supported experimentally, direct causal evidence remains limited regarding the frequency of micronucleus formation in OA, rupture of micronuclear membranes, and whether micronuclear DNA constitutes a major cGAS ligand. More broadly, cGAS studies also indicate that micronuclei and STING activation do not show a simple one-to-one relationship in every cellular context. At present, mtDNA should therefore still be regarded as the best-supported endogenous cGAS stimulus in OA. Nuclear DNA, cytosolic chromatin, and micronuclear DNA are better considered biologically plausible candidate DNA sources that still require *in vivo* validation in OA.

### 2.4 Matrix destruction and cell death establish a sustained reservoir of DAMPs

DNA is not the only danger signal in the OA joint. As the cartilage ECM is progressively degraded, large matrix proteins are cleaved into fragments with distinct biological activities, and some molecules that normally serve structural roles acquire DAMP-like functions (Lambert et al., 2020). Cartilage degeneration therefore has consequences beyond a simple decline in tissue mechanics. The more severe the matrix damage, the larger the pool of endogenous ligands in the joint microenvironment that can be recognized by innate immune systems. These signals can in turn further alter the stress state of surviving cells (Homandbe et al., 1998).

Fibronectin fragments are among the best-studied examples of this mechanism. A 29-kDa amino-terminal fibronectin fragment is present in the synovial fluid of patients with OA and can induce chondrocyte catabolism through multiple pattern-recognition mechanisms. Its effects are not confined to cell-surface receptors. Prolonged exposure of primary human chondrocytes to the 29-kDa fibronectin fragment increases γH2AX and induces cGAS-STING-related responses (Hwang et al., 2019a; Hwang et al., 2019b). Other endogenous alarmins include HMGB1 and S100 proteins. In the synovium of patients with knee OA, HMGB1 shifts from predominantly nuclear localization to a marked cytosolic distribution. Synovial-fluid HMGB1 levels are also higher than in controls and are associated with the severity of synovitis and symptom measures (Ke et al., 2015). S100 calcium-binding proteins A8 and A9 (S100A8 and S100A9) can remain elevated in some OA phenotypes characterized by prominent synovial inflammation. Animal studies show that loss of S100A8/A9 markedly reduces synovial activation and cartilage destruction, while S100A8/A9 expression in early human OA synovium is associated with synovial thickening, cellular infiltration, and subsequent joint damage (van Lent et al., 2012). Not all of these molecules act directly on cGAS. Their importance lies in showing that the OA joint progressively develops a reservoir of danger signals composed of nucleic acids, matrix fragments, and cell-derived alarmins. Different DAMPs may first activate their respective pattern-recognition receptors and then increase new intracellular danger signals through oxidative stress, organelle injury, or DNA damage. Cell death further enlarges this reservoir. Apoptosis, pyroptosis, ferroptosis, and other forms of regulated cell death can occur in OA cartilage, but they differ in their capacity to release intracellular contents. When plasma-membrane integrity is compromised or dead cells are not cleared promptly, DNA, HMGB1, and other intracellular components can escape from normally restricted compartments into the extracellular environment. Increased cell death together with insufficient clearance can therefore shift the local joint environment from episodic danger-signal exposure to a sustained supply of DAMPs.

### 2.5 Aging, obesity, and metabolic stress lower the threshold for danger-signal generation

Healthy cells use DNA repair, antioxidant systems, mitochondrial quality control, and proteostasis to keep damage within a recoverable range. As these protective systems decline, even relatively mild external stimuli may produce substantial DNA damage and organelle injury (Lomeli et al., 2017; López-Otín et al., 2023; Haigis and Yankner, 2010). In other words, a progressive reduction in the stress threshold required to generate danger signals may be an important feature of OA susceptibility.

Aging is closely associated with this change. As cells enter senescence, coordination among DNA-damage repair, mitochondrial homeostasis, and protein quality control gradually deteriorates, making oxidative stress more likely to leave persistent intracellular damage (Minguzzi et al., 2018; Yu and Liu, 2024). Senescence is therefore not only an end-stage phenotype that follows OA progression. It may also establish, in advance, a cellular background in which mtDNA and nuclear DNA are more readily mislocalized. Danger-signal generation in age-related OA should thus be understood as the combined consequence of cumulative damage and declining damage-control capacity rather than as a direct effect of chronological age alone.

Obesity adds metabolic danger stress beyond its contribution to mechanical loading. Saturated fatty acids can directly affect joint cells. For example, palmitate can promote inflammatory responses in human chondrocytes and synovial cells and act together with interleukin-1 beta (IL-1β) to increase cell death and matrix injury (Alvarez-Garcia et al., 2014). Subsequent studies showed that palmitate-associated chondrocyte injury is accompanied by mitochondrial dysfunction and that improving mitochondrial status can reduce the corresponding inflammatory and cartilage-degenerative phenotypes (Sun et al., 2023). Obesity-related OA therefore cannot be explained solely by increased body weight, because circulating lipids and metabolic abnormalities can themselves weaken organelle homeostasis and provide additional sources of danger signals.

Abnormal cholesterol metabolism further suggests a more direct link between metabolic stress and DNA danger signals. A 2025 study found that increased cholesterol in OA joints alters the mitochondrial state of osteocytes in subchondral bone and promotes transfer of osteocyte mitochondria to chondrocytes. Cholesterol stimulation was associated with increased mitochondrial permeability and cytosolic mtDNA accumulation, and mitochondrial nudix hydrolase 8 (NUDT8) was identified as a participant in this metabolic process. Interfering with this pathway reduced cytosolic mtDNA and improved the OA phenotype in mice (Ma et al., 2025). This study is particularly notable because it suggests that metabolic abnormalities can do more than injure individual chondrocytes. Through the exchange of mitochondria and metabolites among different joint tissues, they may also alter the source of danger DNA. Recent work has also begun to examine how adipose tissue in obesity affects chondrocyte mitochondria through circulating or local intercellular carriers. For example, a 2026 study reported that extracellular vesicles derived from obese adipose tissue carry retinol-binding protein 4 (RBP4), which aggravates mitochondrial dysfunction in chondrocytes and promotes OA-related changes (Yang et al., 2026). This field is still developing rapidly, but these findings extend the link between obesity and OA beyond systemic low-grade inflammation to organelle homeostasis and intertissue metabolic communication.

Overall, danger signals in the OA joint do not arise from a single source. Abnormal mechanical stress provides the initial pressure for cellular injury. Mitochondrial dysfunction makes mtDNA the best-supported endogenous DNA source at present, while nuclear-genome damage provides another potential route of DNA supply. At the same time, ECM cleavage and release from damaged cells continuously increase non-nucleic-acid DAMPs, allowing the local microenvironment to persistently stimulate innate immunity. Aging and metabolic abnormalities further weaken the capacity of cells to control these insults, allowing transient danger signals that would normally be repaired or cleared to accumulate. Importantly, these processes do not simply occur in parallel and add together. Mechanical injury can damage mitochondria, matrix fragments can further induce DNA damage, metabolic stress can increase mitochondrial permeability, and declining cellular homeostasis can raise the probability of another round of injury. The joint therefore gradually changes from a tissue environment that can efficiently sequester and clear endogenous danger molecules into a pathological microenvironment that continuously generates immunogenic DNA and DAMPs (Table 1).

**TABLE 1:** Major sources of danger signals in osteoarthritis and their links to cGAS–STING-dependent amplification.

Danger-signal class	How the signal is generated or acts	Connection to cGAS–STING amplification	References
mtDNA	Mitochondrial damage exposes genetic material that is normally segregated from cytosolic sensors	Best-supported endogenous cGAS ligand in OA; cGAS recognition of mtDNA drives the cGAS-cGAMP-STING cascade	Sun L. et al. (2025), Dong et al. (2025), Huang H. et al. (2026)
Aberrantly exposed nuclear DNA	Genome damage can place nuclear DNA outside normal nuclear compartmentalization and make it accessible to cytosolic sensing	A plausible cGAS ligand source, although direct causal evidence in OA is less complete than for mtDNA	Guo et al. (2021), Nagai et al. (2015)
Signals associated with mitochondrial dysfunction	Promote mtDNA injury and escape while favoring inflammasome activity and cell-death responses	Create an upstream permissive state that increases the supply of DNA capable of activating cGAS–STING	Sun L. et al. (2025), Chen S. et al. (2026)
ECM-derived DAMPs	Matrix fragments activate pattern-recognition pathways and can increase cellular stress and DNA damage	Primarily indirect; secondary DNA damage can increase cGAS–STING signaling	Hwang et al. (2019b), Barilla and Carsons (2000)
HMGB1	Acts as an alarmin that stimulates immune cells and inflammatory mediator release	Not a canonical cGAS ligand, but can intensify inflammation and promote secondary danger-signal production	Shao et al. (2023), Li et al. (2011)
S100A8/S100A9	Engage pattern-recognition pathways, recruit inflammatory cells, and promote tissue injury	Facilitate cGAS–STING-related amplification indirectly through inflammatory stress, ROS, and DNA damage	Schelbergen et al. (2014), Schelbergen et al. (2016)
Contents released from dying cells	Loss of membrane integrity exposes normally restricted intracellular components	Replenish potential cGAS ligands and support a damage-inflammation-death-DAMP feedback cycle	Hu K. et al. (2026), Ebata et al. (2023)
EV-borne danger signals	Package and transport multiple bioactive signals between cells while protecting nucleic-acid cargo	mtDNA-containing EVs can transfer DNA directly to recipient cells and re-engage cGAS–STING	Li S. et al. (2026), Sun et al. (2026)
Metabolic stress-related signals	Increase ROS, disturb mitochondrial homeostasis, and lower cellular stress tolerance	Usually act upstream by promoting mitochondrial injury and mtDNA release	Ma et al. (2025), Wang Q. et al. (2026)
Mechanical stress-related signals	Alter ion homeostasis, promote mitochondrial Ca^2+^ overload, and facilitate mtDNA release	Operate near the upstream end of the mechanosensing-mtDNA-cGAS–STING sequence	Sun L. et al. (2025), Dong et al. (2025)

## 3 cGAS-STING-mediated amplification of inflammatory signaling

### 3.1 DNA sensing and initiation of cGAS-cGAMP-STING signaling

Recognition of cytosolic double-stranded DNA by cGAS is the first step in inflammatory amplification. Classical studies showed that binding of mislocalized DNA induces a conformational change in cGAS, which then uses ATP and guanosine triphosphate (GTP) as substrates to synthesize the second messenger 2′3′-cGAMP. cGAMP subsequently binds STING on the endoplasmic reticulum membrane, converting information about DNA damage into an intracellular signal with enzymatic and second-messenger properties. Sun et al. first demonstrated that cGAS is a key DNA sensor mediating type I interferon responses to cytosolic DNA. Knockdown of cGAS markedly attenuated IRF3 activation and interferon beta (IFN-β) production after DNA stimulation (Sun et al., 2013), establishing the basic framework for the current understanding of the cGAS-STING pathway.

Binding of cGAMP to STING does not complete signal transmission. In resting cells, STING is located primarily in the endoplasmic reticulum. After activation, it must exit the endoplasmic reticulum and enter the endoplasmic reticulum-Golgi intermediate compartment and Golgi-associated membrane compartments to efficiently organize downstream signaling complexes. Early cellular studies showed that forced retention of STING in the endoplasmic reticulum markedly weakens DNA-induced innate immune responses. Blocking its export from the endoplasmic reticulum likewise inhibits recruitment of TBK1 to the carboxyl-terminal region of STING and TBK1-mediated phosphorylation of IRF3, indicating that subcellular translocation is itself a critical step in functional STING signaling (Sun et al., 2013; Dobbs et al., 2015; Saitoh et al., 2009). Once STING reaches the appropriate membrane compartment, it provides a signaling scaffold through TBK1 for IRF3. *In vitro* reconstitution studies showed that STING is not merely a passive adaptor; it can interact with both TBK1 and IRF3, spatially positioning IRF3 close to activated TBK1 (Tanaka and Chen, 2012). This spatial organization has important amplifying consequences. cGAS activity triggered by a small amount of cytosolic DNA can be rapidly converted through cGAMP and the STING signaling platform into broad transcription-factor activation, without requiring every downstream effector molecule to contact DNA directly.

In OA cartilage, this canonical DNA-sensing machinery appears to be pathologically enhanced. Studies of human OA cartilage found higher cGAS and STING expression than in normal cartilage. Prolonged stimulation of primary human chondrocytes with the 29-kDa fibronectin fragment commonly found in OA synovial fluid increased γH2AX and activated cGAS-STING-, TBK1-, and IRF3-related signaling. Knockdown of cGAS or STING markedly reduced subsequent inflammatory mediator expression (Hwang et al., 2019b). Guo et al. further observed increased STING expression in human and mouse OA tissues and showed that experimentally increasing or decreasing STING levels produced corresponding changes in chondrocyte inflammation, senescence, apoptosis, and matrix metabolism (Guo et al., 2021). These findings indicate that when persistent abnormal DNA encounters a sensing system with increased responsiveness in OA, the immune output elicited by the same injury burden may change substantially. After cGAS performs the initial conversion of DNA information, STING becomes a branch point for distinct transcriptional programs. This branching capacity is central to the progression of local DNA damage into a complex inflammatory phenotype (Figure 1).

**FIGURE 1:**
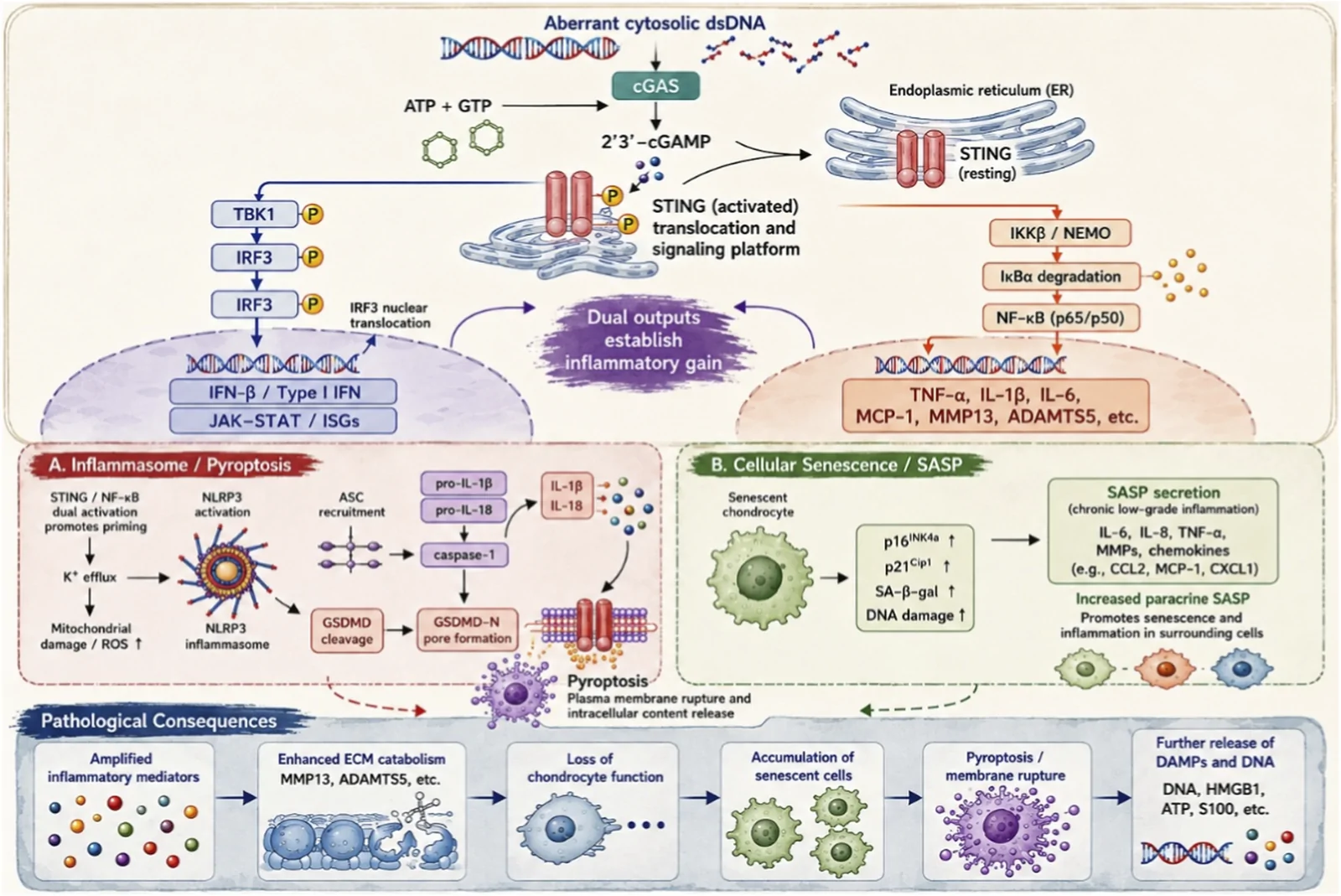
cGAS–STING-dependent amplification of inflammatory and cell-fate responses in osteoarthritis. Cytosolic double-stranded DNA is detected by cGAS, which converts ATP and GTP into 2′3′-cGAMP and activates STING. After trafficking from the endoplasmic reticulum, activated STING supports parallel TBK1–IRF3–type I interferon and IKKβ/NEMO–NF-κB programs. **(A)** Interaction with NLRP3-associated signaling can promote ASC recruitment, caspase-1 activation, IL-1β/IL-18 maturation, GSDMD cleavage, pore formation, and pyroptosis; membrane disruption then releases additional intracellular DAMPs. **(B)** cGAS–STING also contributes to chondrocyte senescence and the SASP, creating sustained paracrine output of inflammatory cytokines, chemokines, and matrix-degrading enzymes. The combined effects increase ECM catabolism, impair chondrocyte homeostasis, expand senescent and inflammatory cell-death programs, and generate new DNA and DAMPs that can re-enter the cycle.

### 3.2 Dual IRF3-type I interferon and NF-κB outputs establish inflammatory gain

The canonical downstream response to STING activation is TBK1-mediated phosphorylation of IRF3. Activated IRF3 dimerizes and enters the nucleus, where it induces IFN-β and other type I interferons and related genes. Type I interferons can further activate Janus kinase (JAK)-signal transducer and activator of transcription (STAT) signaling through autocrine and paracrine mechanisms, inducing a broader set of interferon-stimulated genes and prolonging the transcriptional effects of a single DNA-sensing event. In the general context of innate immunity, this mechanism helps establish a rapid and broad antimicrobial state. In OA, where no pathogen is present but danger DNA may persist, the same mechanism may prolong what would otherwise be transient injury surveillance. Direct evidence indicates that this output branch can be activated in OA chondrocytes. After stimulation of primary human chondrocytes with the 29-kDa fibronectin fragment, expression of interferon alpha (IFN-α), granulocyte-macrophage colony-stimulating factor (GM-CSF), and granulocyte colony-stimulating factor (G-CSF) increased markedly. Knockdown of cGAS or STING reduced TBK1 and IRF3 activation and substantially suppressed production of these inflammatory mediators (Hwang et al., 2019b). These findings demonstrate that the IRF3/IFN-I branch is functionally active under OA-related injury stimulation. However, its cell-type dependence, temporal dynamics, and net effects on tissue repair in OA require more detailed investigation.

Another major STING output in OA cartilage degeneration is NF-κB. NF-κB lies at the convergence of multiple inflammatory and stress signals and regulates inflammatory cytokines, chemokines, and matrix-degrading enzymes (Liu et al., 2017; Marcu et al., 2010). When STING is aberrantly activated, signaling is not confined to the TBK1-IRF3 axis. Basic studies of cGAS-STING signaling have identified functional coupling among NF-κB essential modulator (NEMO), IκB kinase beta (IKKβ), and TBK1. TBK1 and IKKβ can act synergistically to enhance inflammatory transcription, allowing STING to support both IRF3 and NF-κB outputs (Fang et al., 2017). From the perspective of inflammatory amplification, this dual output means that the same DNA-sensing event can establish an interferon-related immune state while also initiating inflammatory transcriptional programs that more closely match the catabolic phenotype of OA cartilage. Guo et al. provided relatively direct evidence for this mechanism in OA. Increased STING expression elevated the catabolic molecules matrix metalloproteinase 13 (MMP-13) and a disintegrin and metalloproteinase with thrombospondin motifs 5 (ADAMTS5), while suppressing aggrecan- and type II collagen-related phenotypes and inducing marked senescence and apoptosis. Inhibition of NF-κB attenuated some of the pathological effects of STING. Reducing STING expression in mice subjected to destabilization of the medial meniscus (DMM) likewise alleviated cartilage degeneration (Guo et al., 2021). These findings indicate that STING activation can directly alter the capacity of chondrocytes to maintain the ECM. Ni et al. further strengthened this link through pharmacological intervention. They found that exogenous itaconate activated nuclear factor erythroid 2-related factor 2 (Nrf2) and inhibited STING-dependent NF-κB signaling, thereby reducing inflammation, ECM degradation, and senescence in IL-1β-stimulated chondrocytes. Intra-articular itaconate also reduced both cartilage destruction and synovitis in mouse OA models (Ni et al., 2022). This reverse-intervention evidence supports an important contribution of the STING-NF-κB axis to chondrocyte inflammation and catabolic phenotypes.

Parallel activation of IRF3/IFN-I and NF-κB gives cGAS-STING substantial signal-amplifying capacity. One branch broadens immune alertness and interferon responses, while the other promotes inflammation and matrix degradation. Both outputs can further alter cellular state through cytokines and stress responses. DNA sensing therefore ceases to be an isolated receptor event and becomes embedded in a larger network that influences cell fate (Figure 1).

### 3.3 cGAS-STING, inflammasomes, and inflammatory cell death form positive feedback

Inflammasomes provide a mode of innate immune amplification that differs from transcriptional activation. Assembly of inflammasomes such as NOD-like receptor family pyrin domain-containing 3 (NLRP3) or absent in melanoma 2 (AIM2) activates caspase-1, promotes maturation of IL-1β and interleukin-18 (IL-18), and induces pore formation and pyroptosis through gasdermin D (GSDMD) (de Vasconcelos and Lamkanfi, 2020). Compared with simply increasing cytokine transcription, pyroptosis causes greater tissue disruption because loss of plasma-membrane integrity allows large amounts of normally intracellular material to enter the extracellular environment (Bai et al., 2025; Feng et al., 2018). In this way, the inflammatory response itself generates new tissue injury and creates conditions for another round of danger sensing.

There is no single fixed connection between cGAS-STING and inflammasomes that applies to all cell types, but experimental evidence shows that the two systems can be directly functionally coupled. In human myeloid cells, Gaidt et al. found that cytosolic DNA can induce a cell-death program upstream of NLRP3 through cGAS-STING. After activation, STING enters lysosome-related compartments, where it can alter membrane permeability and induce potassium (K^+^) efflux, ultimately promoting NLRP3 inflammasome activation (Gaidt et al., 2017). This mechanism shows that STING does not transmit inflammation only through transcription factors. In specific cellular contexts, it can also participate in inflammasome activation through organelle-membrane homeostasis and ionic changes. Cytosolic DNA may also be recognized directly by AIM2, forming the AIM2-apoptosis-associated speck-like protein containing a CARD (ASC)-caspase-1 inflammasome. AIM2-deficiency experiments have long shown that cytosolic double-stranded DNA can promote IL-1β and IL-18 maturation and pyroptosis through this system (Rathinam et al., 2010). For OA, this means that the same pool of abnormally released DNA may not enter only the cGAS-STING pathway. DNA source, cell type, and local ionic and redox conditions may determine whether the response is biased toward interferon signaling or also engages inflammasomes. Once the two DNA-sensing systems are activated in parallel within the same pathological environment, inflammation can progress from transcriptional enhancement to a lytic form of tissue injury.

Evidence for direct coupling between cGAS-STING and pyroptosis in OA is increasing. In 2026, Chen et al. reported in a facet-joint OA model that Asperosaponin VI enhanced PINK1/Parkin-related mitophagy and improved mitochondrial function while suppressing cGAS-STING activity and reducing chondrocyte pyroptosis, ultimately slowing articular cartilage degeneration (Chen S. et al., 2026). This study supports the view that mitochondrial injury, cGAS-STING, and pyroptosis are interconnected pathological modules. However, because the intervention simultaneously improved mitophagy and affected multiple downstream processes, it remains inappropriate to infer that all pyroptosis in OA is directly initiated by cGAS-STING.

Importantly, inflammasomes, pyroptosis, cellular senescence, and cGAS-STING do not form a fixed linear upstream-downstream hierarchy across all cell types and disease stages in OA. These pathological programs can share upstream triggers, including mitochondrial injury, mitochondrial reactive oxygen species (mtROS), aberrant DNA, DNA damage, and disturbed ion homeostasis. In specific cellular contexts, they may also directly intersect and reinforce one another. This crosstalk is particularly important for inflammatory amplification because it can establish a cycle with material feedback. cGAS-STING first enhances inflammation and cellular stress. When inflammasomes and pyroptosis are also engaged, membrane disruption releases DNA and other DAMPs, exposing surrounding viable cells to a greater danger-signal burden. Whether cGAS-STING consistently lies upstream of these processes in different OA phenotypes still needs to be tested using STING- or cGAS-specific genetic models combined with stepwise manipulation of NLRP3, AIM2, and GSDMD (Figure 1).

### 3.4 cGAS-STING-driven cellular senescence and SASP create a chronic amplifier

Senescent cells continuously release inflammatory cytokines, chemokines, proteases, and growth-regulatory molecules through the SASP (Lopes-Pacienci et al., 2019; Ohtani, 2022). In OA, once chondrocytes transition from transient stress to a stable senescent state, they become persistent sources of signals that alter the local microenvironment. cGAS-STING is one important mechanistic link between DNA damage and the SASP. Using multiple models of cellular senescence, Glück et al. found that cytosolic chromatin fragments formed in senescent cells can be recognized by cGAS. Loss of cGAS or STING markedly reduced production of multiple SASP factors and paracrine senescence (Glück et al., 2017). This fundamental mechanism helps explain why DNA damage in OA may not only impair cellular function but also be converted into a persistent inflammatory secretory state.

Direct experimental evidence also links STING to cellular senescence in OA cartilage. Guo et al. found that STING overexpression increased senescence-related phenotypes in chondrocytes, whereas reducing STING expression produced the opposite effect; NF-κB was inhibited. Overexpression of leucine-rich repeat kinase 2 (LRRK2) not only aggravated mitochondrial dysfunction and senescence in chondrocytes but also worsened cartilage damage in the DMM model. Treatment with the STING inhibitor H-151 partially alleviated LRRK2-induced cellular senescence and mitochondrial abnormalities (Zhang et al., 2026a). However, once a stable senescent state is established, epigenetic remodeling, metabolic alterations, persistent DNA-damage responses, and other inflammatory transcriptional programs can become partly independent of cGAS-STING. STING inhibition may therefore reduce some senescence-associated inflammatory outputs without fully reversing an established stable senescent phenotype. This observation suggests that in chronic OA, cGAS-STING-targeted interventions may need to be combined with strategies that reduce persistent DNA damage, modulate the SASP, or eliminate selected senescent cells.

cGAS-STING is an important mechanistic link between DNA damage and the SASP, but this does not mean that cGAS-STING alone is sufficient to initiate stable cellular senescence in every OA context. Senescence in OA is initiated by multiple factors. In this setting, cGAS-STING is more likely to convert persistent abnormal DNA signals into SASP activity and paracrine inflammation, thereby helping maintain and amplify an established senescence-associated inflammatory state (Figure 1).

## 4 Inflammatory amplification spreads from local cells to the whole joint

### 4.1 Chondrocytes shift from injury targets to sources of inflammatory signals

In early OA, chondrocytes maintain ECM homeostasis and directly bear abnormal mechanical loading and changes in the matrix microenvironment. Once chronic stress drives them into catabolic, senescent, or inflammatory states, however, they are no longer merely passive targets of injury. Their secretory profile changes, and the release of proinflammatory mediators, chemokines, matrix-degrading enzymes, and EVs collectively alters the surrounding microenvironment. The consequences of cartilage injury then extend beyond the affected cells, and local cartilage gradually acquires the capacity to export pathological information to other joint compartments (Goldring and Otero, 2011; Varela-Eirín et al., 2022). Direct evidence for this process comes from the effects of chondrocyte-derived EVs on synovial immune cells. Ni et al. found that degenerative cartilage releases more exosome-like vesicles than non-degenerative cartilage. These vesicles can enter synovial tissue and be taken up by macrophages. OA chondrocyte-derived vesicles carrying microRNA-449a-5p (miR-449a-5p) suppress autophagy-related 4 B cysteine peptidase (ATG4B) expression in macrophages, impair autophagy, and increase mitochondrial ROS, thereby enhancing inflammasome activation and mature IL-1β production. In animal experiments, this process was associated with aggravated synovitis (Ni et al., 2019). These findings show that injured chondrocytes can actively alter synovial immune status. Such cartilage-to-synovium communication has strong amplifying potential. Chondrocytes exposed to abnormal stress generate inflammatory responses within cartilage, and once their secreted products alter synovial macrophage states, injury that was initially confined to a small number of chondrocytes gains a new cellular carrier. Macrophage responses to DAMPs can generate much greater inflammatory-mediator output than individual chondrocytes and simultaneously recruit additional immune cells into the synovium (Griffin and Scanzello, 2019) (Figure 2).

**FIGURE 2:**
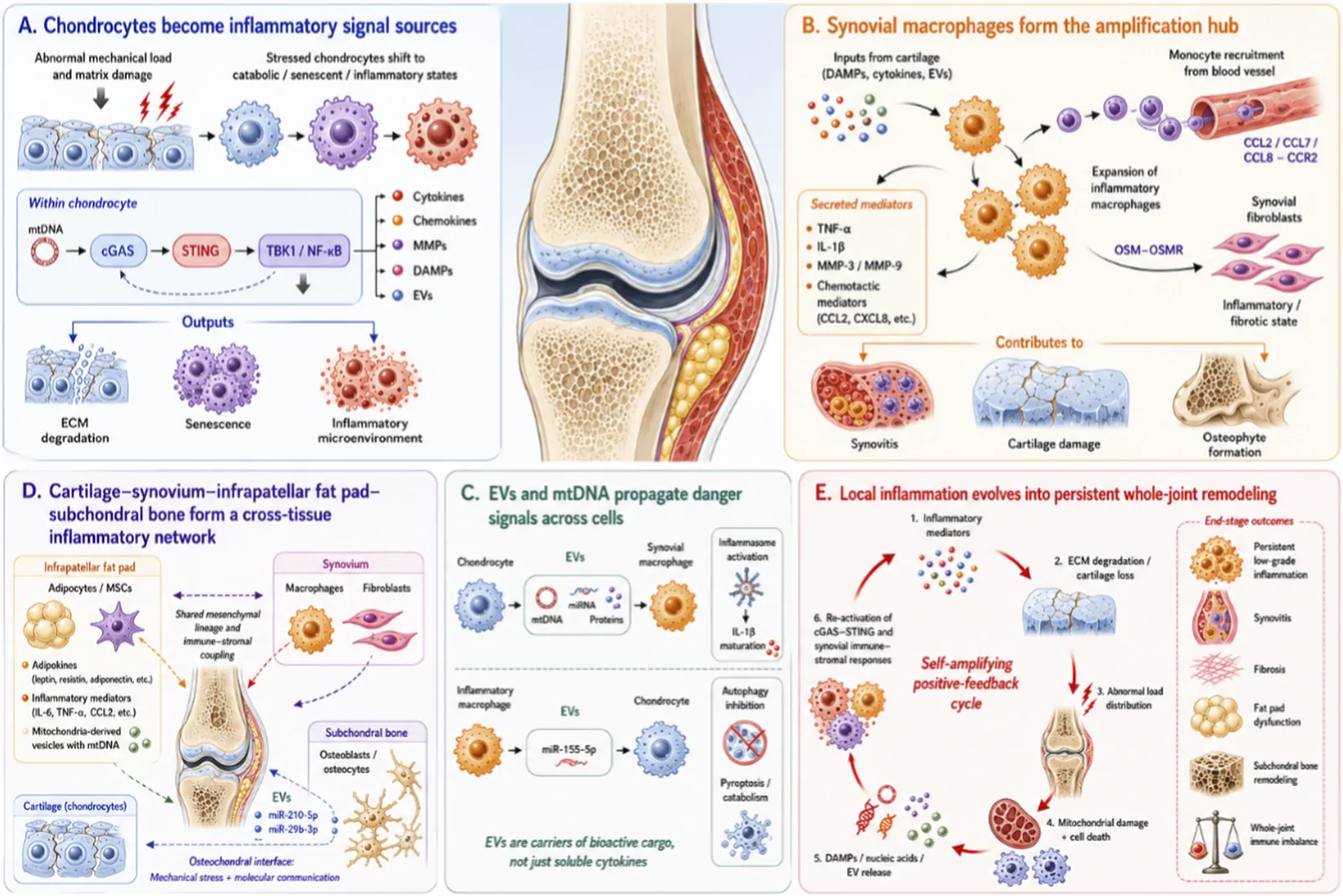
Propagation of the osteoarthritis inflammatory-amplification network across cells and tissues. **(A)** Chondrocytes become inflammatory signal sources. **(B)** Synovial macrophages form the amplification hub. **(C)** EVs and mtDNA propagate danger signals across cells. **(D)** Cartilage–synovium–infrapatellar fat pad–subchondral bone form a cross-tissue inflammatory network. **(E)** Local inflammation evolves into persistent whole-joint remodeling. The model depicts how focal cartilage injury can develop into persistent whole-joint inflammation through reciprocal communication among chondrocytes, synovium, the infrapatellar fat pad, and subchondral bone.

### 4.2 Synovial macrophages form an inflammatory amplification center

Compared with the relatively low metabolic activity of cartilage, the synovium is highly vascularized and contains abundant immune cells. It can rapidly recruit circulating monocytes and expand inflammatory responses. Synovial macrophages therefore serve as an important relay between local cartilage injury and whole-joint inflammation (Scanzello and Goldring, 2012). Synovitis is a common pathological feature of OA, and macrophages, fibroblasts, and infiltrating immune cells within the synovium can establish a persistent inflammatory microenvironment (Sanchez-Lopez et al., 2022; Bondeson et al., 2006). Macrophages are particularly important because they can rapidly sense DAMPs, cytokines, and EVs and have strong secretory and cell-recruitment capacities. Once danger information from cartilage reaches the synovium, macrophages can further translate local tissue injury into chemotactic, inflammatory, and tissue-remodeling signals (Griffin and Scanzello, 2019; Thomson and Hilkens, 2021).

Monocyte recruitment is an important part of this transition. Raghu et al. found elevated levels of C-C motif chemokine ligand 2 (CCL2), C-C motif chemokine ligand 7 (CCL7), and C-C motif chemokine ligand 8 (CCL8), all ligands of C-C motif chemokine receptor 2 (CCR2), in human OA synovial fluid. CCR2-positive cells were abundant in OA synovium and were observed near areas of cartilage erosion. Blocking CCL2/CCR2 signaling markedly reduced macrophage accumulation, synovitis, and cartilage damage in mouse OA (Raghu et al., 2017). These findings show that the spread of joint inflammation depends not only on activation of resident macrophages but also on continuous recruitment of circulating monocytes that expand the immune-cell pool. The effects of increased macrophage numbers are not limited to maintenance of synovitis. Classical animal studies showed that depletion of synovial macrophages early in experimental OA markedly reduced synovial expression of matrix metalloproteinase 3 (MMP-3) and matrix metalloproteinase 9 (MMP-9), as well as MMP-mediated cleavage products in cartilage. MMP-3 deficiency also reduced the incidence of severe cartilage damage (Blom et al., 2007). Another study found that depletion of synovial-lining macrophages substantially reduced osteophyte formation in experimental OA and decreased local responses associated with transforming growth factor beta (TGF-β), bone morphogenetic protein 2 (BMP-2), and bone morphogenetic protein 4 (BMP-4) (Blom et al., 2004). These findings extend the role of macrophages beyond synovial inflammation to cartilage destruction and osteophyte formation, illustrating a characteristic intertissue effect.

Single-cell studies show that OA synovium contains macrophage subsets with distinct origins, transcriptional states, and functions. In 2026, Laouteouet et al. combined mouse lineage tracing, single-cell sequencing of human OA synovium, and imaging mass spectrometry. They found marked expansion of monocyte-derived T-cell immunoglobulin and mucin domain-containing protein 4 (TIM4)-negative macrophages during OA, together with spatial redistribution of tissue-resident macrophages. More importantly, they identified a macrophage subset characterized by low MER receptor tyrosine kinase (MERTK), high cluster of differentiation 48 (CD48), and CCR2 positivity. This subset produced oncostatin M (OSM) and promoted inflammatory and fibrotic states in synovial fibroblasts through communication with the oncostatin M receptor (OSMR) (Laouteouet et al., 2026). This work reveals amplification of inflammatory signaling within the synovium. Monocytes entering the joint do more than produce soluble mediators such as tumor necrosis factor (TNF) and IL-1β. They can also alter the long-term functional state of neighboring fibroblasts. Activated fibroblasts then release chemokines and inflammatory mediators, enabling the synovium to form a self-sustaining immune-stromal network. Macrophages are therefore not simply terminal sources of inflammatory mediators; they act as intermediaries that convert cartilage-injury signals into synovial tissue remodeling (Figure 2).

### 4.3 Extracellular vesicles and mtDNA mediate intercellular propagation of danger signals

EVs include multiple types of membrane-bound particles generated through different biogenetic pathways. Their cargo can include proteins, lipids, messenger RNA (mRNA), microRNA (miRNA), and DNA. Compared with free cytokines, an important feature of EVs is their ability to deliver a set of biologically active molecules as combined cargo to recipient cells while providing some membrane-mediated protection for enclosed nucleic acids (Palanisamy et al., 2010; Picon et al., 2023). EVs in the OA joint arise from diverse sources, including chondrocytes, synovial fibroblasts, macrophages, mesenchymal stromal cells, adipose-tissue cells, and bone cells, giving them a natural capacity to connect different joint compartments (Fan et al., 2022). Recent work also shows that small EVs derived from OA synovial fibroblasts can be taken up by chondrocytes and enhance catabolic responses involving interleukin-6 (IL-6), interleukin-8 (IL-8), MMP-3, and MMP-13, indicating that synovium-to-cartilage information transfer can also occur through vesicles (Asghar et al., 2024).

A 2025 study found that both the proportion of EVs and the mtDNA content of synovial fluid were increased in patients with OA compared with trauma patients. The study further identified mitochondria-derived vesicles originating from mesenchymal stromal cells in the infrapatellar fat pad. These vesicles could fuse with chondrocytes and deliver exogenous mtDNA to recipient cells. This was followed by mitochondrial dysfunction, suppression of cartilage-matrix synthesis, and enhanced inflammatory signaling involving cGAS-STING, TBK1, and NF-κB (Li S. et al., 2026). This study provides a material basis for cGAS-STING-associated inflammatory spread that differs from cytokine-mediated paracrine signaling. Mitochondrial injury in one cell no longer implies that danger DNA can be sensed only within that cell. mtDNA can be packaged into specific vesicles and transferred to another cell, allowing the recipient to receive DNA danger information even when its own genome has not first undergone severe damage.

EV-mediated inflammatory communication is also clearly bidirectional. Chondrocyte-derived vesicles can enter synovial macrophages and promote IL-1β maturation, while inflammation-stimulated macrophages can alter chondrocytes in the opposite direction through EVs. Studies show that EVs derived from inflammatory macrophages can induce noncanonical pyroptosis in chondrocytes and enhance cartilage catabolism (Ebata et al., 2023). Another study found that inflammatory synovial macrophages can deliver miR-155–5p through EVs, thereby suppressing autophagy in chondrocytes through glycogen synthase kinase 3 beta (GSK-3β)/mechanistic target of rapamycin complex 1 (mTORC1)-related signaling. Selective reduction of miR-155 in these macrophages alleviated cartilage injury and synovitis in experimental OA (Zhao S. et al., 2026). Together, these findings show that cartilage and synovium do not have a fixed starting point for signaling. Early mechanical injury may first occur in cartilage, after which cartilage-derived outputs alter synovial immune states. Once synovitis develops, EVs produced by macrophages and fibroblasts can act back on cartilage, turning inflammatory spread into a bidirectional cycle. mtDNA transfer directly connects this cycle to cGAS-STING, whereas miRNAs, proteins, and other EV cargo influence autophagy, metabolism, and cell death through different routes.

Moreover, the pathological effects of EVs cannot be attributed simply to a single mtDNA cargo. OA-associated EVs typically carry miRNAs, proteins, lipids, mRNA, DNA, and varying amounts of mitochondrial components. These cargoes can independently influence autophagy, mitochondrial homeostasis, inflammasomes, metabolism, and matrix remodeling. EV-associated mtDNA is important because it provides a direct mechanism for intercellular transfer of DNA danger signals and cGAS-STING activation in recipient cells. Nevertheless, the net pathological effect of a specific EV population should be understood as the combined result of multiple cargoes and the state of the recipient cell rather than being attributed entirely to mtDNA transfer (Figure 2).

### 4.4 Cartilage, synovium, infrapatellar fat pad, and subchondral bone form an intertissue inflammatory network

When cytokines, chemokines, and EVs are repeatedly exchanged within synovial fluid, the spatial extent of inflammation is no longer confined to cartilage and synovium. The infrapatellar fat pad lies adjacent to both structures, while subchondral bone shares mechanical loading with cartilage across the osteochondral interface. These anatomical relationships provide routes for inflammatory signaling to spread further.

The infrapatellar fat pad was previously regarded mainly as an adipose structure that fills the joint cavity and buffers mechanical stress, but increasing evidence indicates that it has active immune and secretory functions (Clockaerts et al., 2010). In patients with OA, the infrapatellar fat pad contains macrophages, adipocytes, fibroblast-like cells, and mesenchymal stromal cells and can produce a range of adipokines, inflammatory mediators, and EVs (Klein-Wieringa et al., 2011). Because it is anatomically continuous with the synovium, pathological changes in the infrapatellar fat pad do not occur independently and may form, together with the synovium, an important immunometabolic compartment in the anterior joint. Single-cell studies provide additional evidence for this functional integration at the cellular level. Li et al. performed single-cell analyses of synovium and infrapatellar fat pad from normal and OA mice and found that multiple mesenchymal cell populations in both tissues share progenitors positive for dipeptidyl peptidase 4 (DPP4). After OA injury, related progenitors and synovial-lining fibroblasts could transition toward a myofibroblast state and persist within the thickened synovium (Li et al., 2024). Human single-cell and single-nucleus RNA sequencing likewise suggests shared mesenchymal lineages and close communication between the infrapatellar fat pad and synovium, further supporting the view that they function as a highly coupled tissue unit (Tang et al., 2024). This unit can influence cartilage through several routes. Adipokines and inflammatory mediators released from the infrapatellar fat pad can alter chondrocyte metabolism, while specific mitochondria-derived vesicles from infrapatellar-fat-pad mesenchymal stromal cells can directly deliver danger DNA into chondrocytes (Li S. et al., 2026). Thus, within the cGAS-STING framework, the fat pad is not merely a source of systemic metabolic context; it can also act as a local source of DNA danger information. At the same time, inflammatory mediators generated by synovitis and cartilage injury can alter immune and stromal cell states in the infrapatellar fat pad, creating a continuous local microenvironment across the three tissues.

Subchondral bone participates in this network through different routes. Early subchondral-bone remodeling in OA can occur before overt cartilage defects, and abnormal bone turnover, local sclerosis, vascular changes, and osteocyte mechanosensing can all alter the biological state of the osteochondral unit (Funck-Brentano and Cohen-Solal, 2015; Zhang and Wen, 2021). This connection was previously explained mainly by mechanical transmission, but EV studies show that subchondral bone can directly transmit molecular information to cartilage. Wu et al. isolated osteoblast-derived EVs from sclerotic and non-sclerotic regions of human OA subchondral bone and found that EVs from sclerotic regions were taken up by chondrocytes, promoted catabolism, and suppressed cartilage-specific phenotypes. Enriched miR-210–5p altered chondrocyte bioenergetics (Wu et al., 2021). A 2025 study further incorporated mechanical stress into bone-to-cartilage EV communication. Liu et al. found that mechanical stimulation of subchondral osteocytes increased osteocyte EV release. Among their cargoes, miR-23b-3p acted on OTU deubiquitinase 4 (OTUD4) in chondrocytes and disrupted mitophagy, ultimately enhancing cartilage catabolism. Manipulating miR-23b-3p in either osteocytes or chondrocytes alleviated cartilage degeneration in mice (Liu et al., 2025). These findings connect mechanosensing in subchondral bone, EV transfer, and mitochondrial homeostasis in chondrocytes. The osteochondral interface is therefore not merely a mechanically coupled structure; it is also a molecular conduit through which organelle injury and inflammatory susceptibility can spread.

Current evidence remains insufficient to determine whether cGAS-STING is as important in different subchondral-bone cell populations as it is in chondrocytes. In experimental OA, nitisinone reduces chondrocyte inflammation and ECM degradation and inhibits cGAS-STING/NF-κB-related signaling while also reducing osteoclast formation and subchondral-bone remodeling (Yang T. et al., 2024). These parallel improvements suggest that DNA-sensing networks may be linked to shared pathological changes across the osteochondral unit. However, they do not by themselves establish that osteocytes, osteoblasts, or osteoclasts in subchondral bone are directly driven by intracellular cGAS-STING. Future cell-specific genetic models are needed to distinguish indirect improvements in bone remodeling caused by STING inhibition in cartilage from direct contributions of STING signaling within bone cells.

Viewed as a whole, cartilage, synovium, the infrapatellar fat pad, and subchondral bone are not four tissues affected in parallel. Cartilage-derived signals can activate synovial macrophages, while synovial immune and stromal cells can feed back to promote cartilage catabolism. The infrapatellar fat pad shares some cellular lineages with the synovium and can influence cartilage through metabolic factors and EVs. Subchondral bone converts mechanical stress into molecular communication that crosses the osteochondral interface. Multi-tissue single-cell atlases have observed concurrent increases in inflammatory macrophages, osteogenic stromal cells, and destructive chondrocytes in OA, together with active intertissue communication, consistent with these experimental findings (Raut R. et al., 2025). Once this communication becomes persistent, inflammatory spread enters a stage that is more difficult to reverse. Disease maintenance no longer depends on whether the initial mechanical injury persists, because structural changes themselves create new mechanical abnormalities, while inflammation and cellular injury continuously provide new molecular stimuli (Figure 2).

### 4.5 From local inflammation to persistent whole-joint remodeling

A major feature of chronic OA is the gradual loss of the joint’s ability to return injury responses to homeostasis. Early in disease, inflammation triggered by a single mechanical stimulus may still be reversible. As cartilage matrix is lost, synovial cellular composition changes, the fat pad becomes fibrotic, and subchondral bone is remodeled, the original joint architecture and cellular ecology are progressively reshaped. Even if a particular inflammatory episode subsides temporarily, new mechanical abnormalities and tissue communication can regenerate danger signals. The disease therefore becomes characterized by recurrent activation rather than by a single unchanging inflammatory process.

This transition first appears as increasing interdependence between inflammation and structural damage. Inflammatory mediators and proteases released by synovial macrophages accelerate cartilage-matrix degradation, and macrophage-depletion experiments show that altering a single synovial cell population can markedly reduce MMP-mediated matrix damage in distant cartilage (Blom et al., 2007). As cartilage thickness and matrix organization decline, load distribution becomes more uneven, exposing the remaining chondrocytes and subchondral bone to higher local mechanical stress. Mechanical stress can then promote osteocyte EV release and mitochondrial injury in chondrocytes, allowing structural degeneration itself to become the starting point for another round of molecular injury (Liu et al., 2025). A second maintenance factor is the stable reprogramming of inflammatory and stromal cell states. Monocytes recruited into the synovium can form specific macrophage subsets and drive fibroblasts toward inflammatory and fibrotic states through signals such as OSM (Laouteouet et al., 2026). The transition of shared progenitor populations from the infrapatellar fat pad and synovium toward myofibroblasts after OA injury likewise indicates that tissue remodeling is not controlled solely by short-lived cytokines but can be encoded in relatively stable cellular states (Li et al., 2024). Once such changes in cellular composition and spatial structure are established, the threshold for subsequent responses to mechanical or metabolic stimuli also changes. A third key factor is failure of damage clearance and inflammatory resolution. After normal tissue injury, dead cells and cellular debris should be removed promptly, and inflammatory cells should exit their activated state. Macrophage heterogeneity, accumulation of senescent cells, and metabolic abnormalities in OA may impair these homeostatic functions. In obesity-associated OA, for example, reduced growth arrest-specific 6 (GAS6) is associated with impaired efferocytosis by synovial macrophages and more pronounced synovitis, suggesting that ineffective clearance of dead cells can itself prolong DAMP exposure (Yao et al., 2023). When cell death exceeds clearance capacity, sufficient material to activate innate immunity may remain in the joint even after the initiating injury has weakened.

When these processes are integrated, the core of whole-joint inflammatory amplification is not an unlimited rise in the concentration of any single cytokine, but the progressive coupling of qualitatively different feedback mechanisms. Inflammatory mediators alter chondrocyte and synovial-cell states. Structural damage changes joint mechanics, and abnormal mechanics again cause mitochondrial and cellular injury. Cell death and EV release increase the burden of DAMPs and nucleic acids, while immune-stromal networks in the synovium and fat pad prolong these signals. Subchondral-bone remodeling further alters stress and molecular communication within the osteochondral unit. The increases in inflammatory macrophages and osteogenic cells, together with decreases in regenerative stromal cells observed in cross-tissue single-cell analyses, reflect the progression of OA from local injury to whole-joint cellular ecological imbalance. At this stage, cGAS-STING is better positioned as a network node than as the sole disease driver. Current evidence clearly shows that aberrant mtDNA can activate this pathway within chondrocytes and can also be transferred from infrapatellar-fat-pad-derived cells to chondrocytes through specific EVs, thereby reinitiating DNA sensing (Li S. et al., 2026). At the same time, extensive cytokine, miRNA, and metabolic communication among chondrocytes, macrophages, and synovial fibroblasts occurs independently of cGAS-STING. A more plausible model is that cGAS-STING participates in one class of DNA danger-signal propagation with strong amplifying potential and is nested within other immune and tissue-remodeling pathways. This model captures its hub-like value in OA without reducing a complex whole-joint disease to a single molecular pathway (Figure 2).

## 5 Therapeutic strategies targeting cGAS-STING

### 5.1 Direct pharmacological inhibition of core cGAS-STING nodes

Drugs can act directly on cGAS or STING to reduce cGAMP production or block STING signal transduction. At the pharmacological level, these strategies target core proteins in the DNA-sensing pathway and have a clear theoretical advantage: they can interrupt signaling before multiple downstream inflammatory programs diverge. Although cGAS and STING belong to the same pathway, they represent different levels of pharmacological intervention. Inhibiting cGAS primarily limits conversion of aberrant DNA into cGAMP and therefore preferentially blocks DNA-dependent input. STING inhibition acts further downstream and can simultaneously weaken multiple STING-dependent transcriptional and cellular-stress programs. Both approaches are mechanistically plausible in OA characterized by persistent mtDNA leakage, but direct pharmacological evidence in OA currently focuses more on STING (Shin et al., 2023; Zhu et al., 2024). Specific inhibition of cGAS itself has been studied less systematically in primary OA. Several small-molecule cGAS inhibitors have entered medicinal-chemistry optimization, but they have been investigated mainly in autoinflammatory diseases and other disorders involving DNA sensing (Hao et al., 2026).

The protective effects of STING inhibition in OA are supported by multiple models. In the DMM mouse model of OA, the STING inhibitor C-176 has been used to demonstrate pathway tractability. Aerobic exercise reduced cGAMP generation and cGAS-STING activity and improved cartilage degradation, inflammation, and chondrocyte apoptosis. C-176 alone produced similar chondroprotective effects, whereas addition of a STING agonist weakened the benefits of exercise. This pharmacological cross-validation moves STING beyond the status of a molecular marker associated with disease changes and closer to a functional node capable of altering OA progression (Mao et al., 2026).

H-151 is another STING-inhibitory tool commonly used in experimental research. Early pharmacological studies showed that this class of covalent STING inhibitors can act on cysteine residues in the transmembrane region, interfering with activation-associated lipid modification and subsequent signaling-complex formation (Haag et al., 2018). In OA-related chondrocyte experiments and animal models, H-151 partially reversed LRRK2-induced chondrocyte senescence and mitochondrial dysfunction, supporting the potential of STING inhibition to modify senescence-associated cellular phenotypes in OA (Zhang et al., 2026a). A contemporaneous study of knee OA pain also included an H-151 intervention group and linked modulation of cGAS-STING to vascular endothelial growth factor A (VEGFA)-associated nociception, inflammation, and cartilage lesions (Hu E. et al., 2026).

Understanding of the pharmacological mechanisms of STING continues to evolve. Recent studies of human STING suggest that palmitoylation at cysteine residue 91 (Cys91) is not indispensable in every human STING signaling context and that other cysteine modifications and oligomerization states also contribute to regulation (Chan et al., 2025). Future STING inhibitors for OA may therefore need to be optimized for human STING structure, common genetic variants, and signaling characteristics in different tissue cell types rather than simply reproducing the mechanisms of early tool compounds. At present, however, direct cGAS-STING inhibition in OA is still supported mainly by cell experiments and animal models. No randomized human OA trial has yet centered on a cGAS- or STING-specific inhibitor. For chronic OA, reducing the abnormal DNA sources that sustain pathway activation may offer a gentler therapeutic window than pursuing increasingly potent systemic STING inhibition (Figure 3).

**FIGURE 3:**
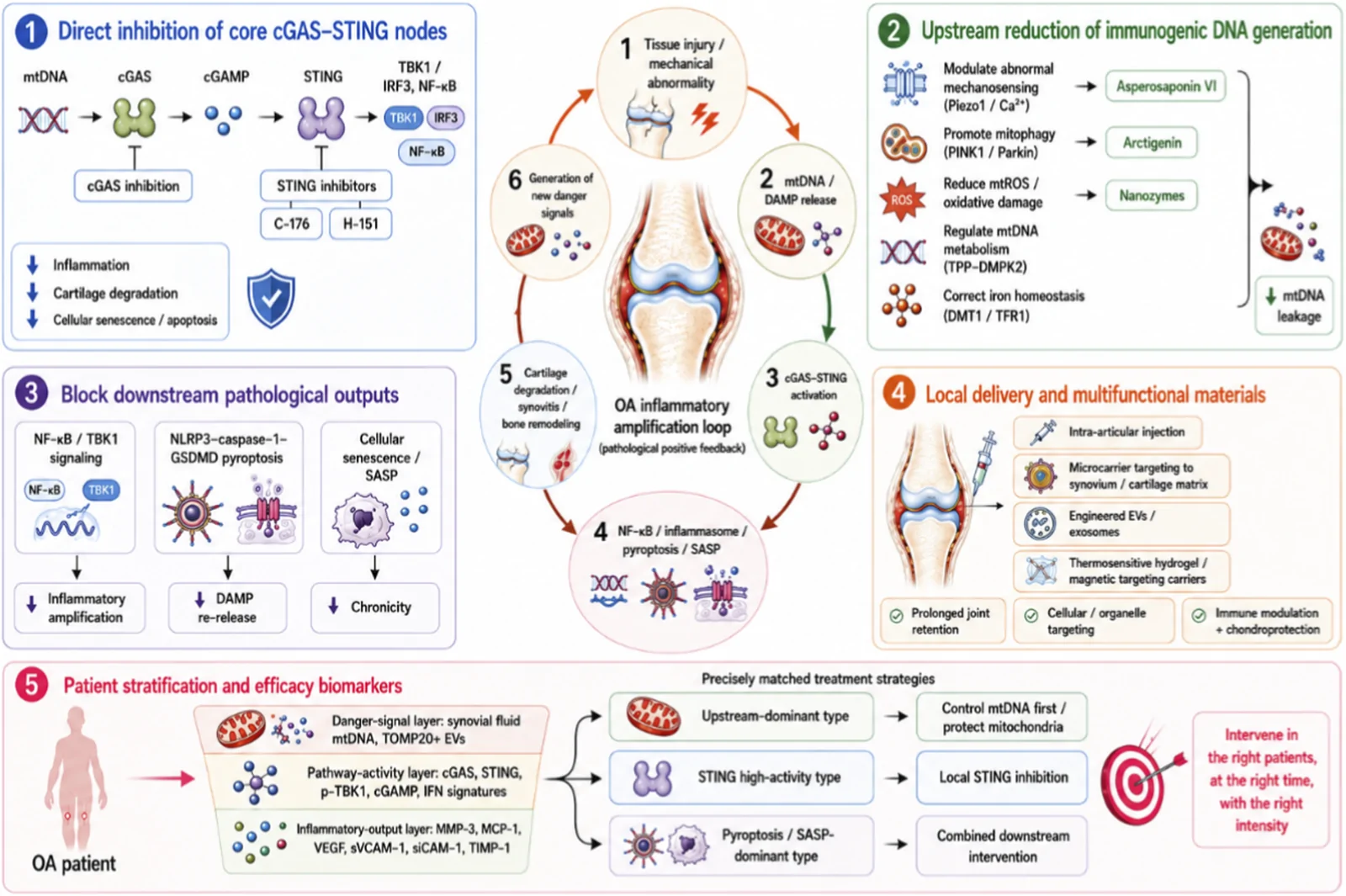
Therapeutic intervention points along the cGAS–STING inflammatory-amplification axis in osteoarthritis. The central loop links tissue injury and abnormal loading to mtDNA/DAMP release, cGAS–STING activation, NF-κB and inflammasome signaling, pyroptosis/SASP, structural damage, and generation of new danger signals.

### 5.2 Indirect attenuation by limiting upstream immunogenic DNA generation

Unlike pharmacological strategies that act directly on cGAS or STING proteins, upstream restriction of immunogenic DNA reduces the DNA input that chronically activates cGAS-STING, for example, by lowering mtROS or limiting mtDNA generation. It therefore represents an indirect form of pathway inhibition. Reducing danger-DNA generation does not require complete shutdown of cellular DNA sensing. Instead, the goal is to restore mitochondrial quality control so that DNA normally sequestered within mitochondria no longer enters the cytosol continuously. For OA, which often requires long-term management, this approach may be closer to restoring cellular homeostasis.

Mechanosensing is an upstream step that can be targeted early. Studies have shown that Ca^2+^ influx and mitochondrial Ca^2+^ overload caused by abnormal mechanical stimulation are closely linked to mtDNA release, whereas Piezo1 deletion reduces subsequent cGAS-STING activation and cartilage degeneration (Sun L. et al., 2025; Yan Z. et al., 2026). The value of this strategy is that it moves the therapeutic target upstream of DNA leakage. Particularly in OA subtypes dominated by abnormal mechanical loading, modulating pathological mechanosensing may reduce both mitochondrial injury and other catabolic programs. However, because Piezo1 contributes to normal mechanosensing, an optimal intervention would more likely restrain chronic overactivation than permanently eliminate channel function.

Moving further upstream within mitochondria, restoration of mitophagy may reduce the long-term accumulation of damaged organelles. Studies of Asperosaponin VI in chondrocytes and animal models of facet-joint OA found that the compound enhanced PINK1/Parkin-related mitophagy, improved mitochondrial status, reduced cGAS-STING activation and chondrocyte pyroptosis, and ultimately delayed OA progression in the facet joint (Chen S. et al., 2026). Asperosaponin VI has multiple pharmacological effects, so its benefits cannot be attributed entirely to reduced mtDNA. Nevertheless, the study demonstrates an important therapeutic principle: improving mitochondrial quality control can simultaneously reduce the source of danger signals and downstream inflammatory cell death without directly targeting STING itself.

mtDNA production and release are also subject to post-transcriptional regulation. TTP is reduced in OA cartilage, and TTP can bind the 3′untranslated region of CMPK2 mRNA and promote its degradation, thereby limiting CMPK2-associated cytosolic mtDNA accumulation. Loss of TTP aggravates cartilage degeneration, whereas overexpression is protective. The investigators further used arctigenin to enhance TTP-related activity, reducing CMPK2, mtDNA release, and cGAS-STING/NF-κB activity in mice and improving OA pathology (Huang H. et al., 2026). This study broadens the concept of upstream therapy because danger-DNA levels are not determined solely by whether mitochondria rupture; they can also be altered through regulation of mtDNA metabolism and homeostasis.

Reducing mitochondrial ROS is another related approach. mtROS can damage mtDNA and also affect mitochondrial membrane permeability and inflammasome activation. If ROS can be scavenged at the appropriate subcellular site, both mtDNA oxidation and cytosolic leakage may be reduced. A 2025 study using a membrane-biomimetic nanozyme decreased mtDNA leakage in chondrocytes through mitochondria-targeted scavenging of mtROS and suppressed cGAS-STING-NF-κB-related inflammation, producing chondroprotective effects in OA mice (Fan et al., 2025). A 2026 study of a MXene nanozyme simultaneously restored mitochondrial membrane potential, promoted mitophagy, and inhibited mtDNA-driven cGAS-STING inflammation, further showing that restoration of mitochondrial homeostasis can be integrated with cartilage regeneration and immune modulation (Li T. et al., 2026). Two studies published in 2024 further showed that increased iron uptake mediated by divalent metal transporter 1 (DMT1) or transferrin receptor 1 (TfR1) leads to iron accumulation and oxidative stress, causing additional mitochondrial injury and promoting mtDNA release and cGAS-STING-mediated inflammatory responses. Silencing either transporter reduced iron accumulation, promoted mitophagy, suppressed mtDNA-cGAS-STING inflammation, and alleviated cartilage degeneration (Wang et al., 2024; Liu et al., 2024).

Upstream intervention and direct STING inhibition are not competing approaches. In patients with earlier-stage disease and relatively reversible mitochondrial dysfunction, reducing the source of injury may more closely address disease causation. Once DNA sensing and inflammatory feedback are established, however, restoration of mitochondrial function alone may not terminate an existing immune response quickly enough. Short-course STING inhibition or downstream anti-inflammatory treatment may then be more appropriate as part of a combination strategy. These stage-dependent differences suggest that effective therapeutic combinations should be selected according to whether the inflammatory amplification loop is in its initiation, maintenance, or tissue-remodeling phase (Figure 3).

### 5.3 Blocking downstream inflammatory feedback and cell death

When cGAS-STING sustains tissue injury mainly through NF-κB, inflammasomes, pyroptosis, or the SASP, selectively limiting these pathological outputs may provide a wider therapeutic safety window. This approach preserves some upstream danger-sensing function while reducing the execution programs that directly drive cartilage-matrix degradation and paracrine inflammation. However, downstream nodes also receive input from Toll-like receptors (TLRs), nucleotide-binding oligomerization domain (NOD) receptors, cytokine receptors, and metabolic stress. Their advantage is broad coverage, whereas their disadvantage is reduced target specificity. Overall, downstream therapy is best viewed as selective attenuation of pathological output.

NF-κB is one of the downstream targets supported by relatively direct evidence in OA. Ni et al. found that exogenous itaconate activated Nrf2 and reduced STING-dependent NF-κB activity, simultaneously decreasing inflammation, ECM degradation, and cellular senescence in IL-1β-stimulated chondrocytes. Intra-articular itaconate improved cartilage structure, reduced synovitis, and altered inflammatory macrophage states in mouse OA models (Ni et al., 2022). Liu et al. reported that a cepharanthine-loaded tetrahedral framework nucleic acid platform (tFNA@Cep) promoted macrophage polarization toward an anti-inflammatory M2 phenotype while suppressing the cGAS-STING-NF-κB pathway and ROS generation. In a mouse model of temporomandibular joint osteoarthritis (TMJOA), tFNA@Cep improved cartilage thickness and subchondral-bone architecture and restored expression of type II collagen (Col II) and runt-related transcription factor 2 (RUNX2). Conditioned medium from the modulated macrophages also promoted anabolic gene expression in chondrocytes, suggesting that targeting the cGAS-STING inflammatory axis while reshaping macrophage function may combine inflammatory control with osteochondral repair.

Inflammasomes and pyroptosis constitute another intervention level. OA studies have shown that NLRP3-associated pyroptosis promotes cartilage injury, and studies from 2025–2026 continue to identify candidate strategies that improve experimental OA by limiting NLRP3-, caspase-1-, and GSDMD-related programs (Zhou and Huang, 2025; Zh et al., 2025). Given that cGAS-STING and inflammasomes can share upstream conditions such as mtDNA and ROS, blocking inflammatory cell death in OA subtypes with prominent pyroptosis may reduce renewed DAMP release and thereby weaken the material feedback that sustains the amplification loop. However, evidence remains insufficient to show that all therapeutic effects of NLRP3 targeting depend on cGAS-STING. These systems should therefore be treated as intersecting rather than strictly serial therapeutic targets.

Cellular senescence offers another entry point that is more relevant to chronic disease stages. Unlike pyroptosis, which rapidly releases intracellular contents, senescent cells can survive for long periods while maintaining the SASP, substantially prolonging inflammation. Anti-senescence strategies for OA have expanded from senolytics that directly eliminate senescent cells to senomorphics that suppress the SASP, antioxidant materials, metabolic modulation, and EV-based therapies (Afzal et al., 2025). In OA with high cGAS-STING activity, such approaches may be particularly complementary. STING contributes to senescence and SASP formation, but once stable senescence is established, some epigenetic and metabolic programs may continue to sustain abnormal secretion even after short-term STING reduction (Kong et al., 2026; Liu et al., 2026). Combining reduction of DNA damage with SASP restriction may therefore be more likely to weaken chronic feedback than targeting either end alone, although this combination still requires direct experimental validation (Figure 3).

### 5.4 Local delivery and multifunctional materials to widen the therapeutic window

OA has an anatomical feature that may favor cGAS-STING-targeted therapy: pathology is concentrated within specific joints. Compared with systemic autoinflammatory diseases, OA of the knee, hip, or temporomandibular joint can, at least in principle, be treated locally to increase exposure of diseased tissues while reducing systemic cGAS-STING inhibition. Conventional intra-articular injection has already shown the feasibility of locally modulating STING-related inflammation. For example, intra-articular delivery of agents such as itaconate and nitisinone can improve cartilage degeneration and synovitis in mouse OA (Ni et al., 2022; Yang T. et al., 2024; Lu et al., 2024). However, rapid clearance of small molecules from synovial fluid, the diffusion barrier created by dense cartilage ECM, and the adherence burden of repeated injections make simple increases in local dosing frequency unattractive as a long-term strategy.

Recent biomaterial strategies have begun to integrate prolonged intra-articular retention with reduction of mtDNA sources. Mitochondria-targeted nanozymes extend spatial precision to the subcellular level. A recently developed MW@GeSe@HMP nanocomposite hydrogel integrates a cartilage-targeting and mitochondria-protective chimeric peptide, a two-dimensional germanium selenide (GeSe) nanozyme, and MMP-13-responsive release. By scavenging ROS, improving mitochondrial function, and inhibiting cGAS-STING signaling, the platform cooperatively disrupts the oxidative stress-inflammation-senescence network, reduces cartilage-matrix degradation, and delays OA progression (Zhang Z. et al., 2026). RAPA@MPB-MMP9 nanoparticles developed in 2024 use cell selectivity and mitochondrial targeting to reduce mtROS and mtDNA release from synovial macrophages. They also reduce GSDMD-dependent pyroptosis and promote mitophagy while modulating cGAS-STING activation, thereby suppressing synovial inflammation and cartilage degradation and slowing OA progression (Qi et al., 2024). These multifunctional materials reflect an important shift in OA therapeutic strategy. Simply loading more anti-inflammatory drug into a nanocarrier has limited value. Greater potential lies in using pathological-environment responsiveness and organelle targeting so that treatment is released preferentially where ROS levels are high, mitochondrial injury is prominent, or inflammation is active. This approach can combine reduction of danger-DNA sources with protection of the cartilage matrix and may also limit exposure of normal cartilage and systemic immune cells.

EVs may also serve as local therapeutic carriers. Engineered EVs can deliver mitochondrial quality-control proteins, mRNA, or other regulatory molecules into chondrocytes. OA studies have shown that mesenchymal stromal cell (MSC)-derived EVs enriched in functional mitochondrial components can improve mitochondrial status and senescence in stressed chondrocytes and delay experimental OA. Recent work on engineered EVs further highlights the possibility of improving cartilage targeting and cargo-loading efficiency (Li et al., 2025; Zhang et al., 2026c). Other studies have combined engineered exosomes with injectable thermosensitive hydrogels to prolong local release of cargo that improves mitochondrial quality control (Ma et al., 2024).

The more complex the material system, however, the greater the barriers to translation from laboratory studies to clinical use. First, intra-articular delivery requires a balance between sufficiently long local retention and controllable clearance. Insufficient retention reduces effective exposure, whereas prolonged accumulation requires clarification of *in vivo* degradation, metabolism, and potential tissue toxicity (Gao et al., 2022). Second, nanomaterials and nanozymes still require systematic evaluation of long-term biocompatibility, immunogenicity, degradation products, intra-articular distribution, and possible spread to extra-articular tissues. Third, multicomponent hydrogels, responsive nanoplatforms, and engineered EVs face challenges in establishing manufacturing standards, batch consistency, scale-up, quality control, and potency assays (D'Arrigo et al., 2024). For EVs in particular, substantial heterogeneity in source cells, isolation and purification methods, particle subpopulations, cargo composition, and effective dose limits cross-study comparison and the development of regulatory standards (Karoichan et al., 2024). Dense cartilage ECM also restricts penetration, and rapid clearance of some carriers from synovial fluid may prevent ideal joint retention and target-cell exposure. Future studies must therefore do more than show that these platforms reduce cGAS-STING activity in animal models. They must define *in vivo* degradation trajectories, pharmacokinetics and tissue distribution, long-term toxicity, joint residence time, manufacturing reproducibility, and feasibility of large-scale production before genuine clinical translation can be assessed (Soh et al., 2024; Kalinkovich and Livshits, 2025) (Figure 3).

### 5.5 From target engagement to patient stratification and response biomarkers

The marked heterogeneity of OA may be the most important issue to resolve before cGAS-STING-targeted therapy can advance toward the clinic. OA phenotypes dominated by mechanical overload, senescence, metabolic abnormalities, or inflammation may share some pathological processes, but this does not mean that every patient has the same degree of mtDNA leakage or STING activation. In patients without substantial pathway activity, a STING inhibitor may bind its target effectively yet still fail to produce a clear disease-modifying effect (Wang W. et al., 2026). An ideal translational pathway would first identify patients with relatively high levels of DNA damage, mitochondrial DAMP burden, and cGAS-STING activity, and would then use these measures to determine whether treatment actually disrupts the pathological loop.

Among candidate biomarkers at the danger-signal level, mtDNA has strong mechanistic relevance. Studies of synovial-fluid EVs from patients with OA found higher proportions of EVs positive for translocase of outer mitochondrial membrane 20 (TOMM20) and greater EV-associated mtDNA content than in controls. Specific mitochondria-derived vesicles could transfer mtDNA to chondrocytes and activate cGAS-STING-related inflammation (Li S. et al., 2026). EV-associated mtDNA therefore lies closer to the upstream pathway than measurements of general inflammatory mediators and suggests that analysis of mitochondria-derived EVs in synovial fluid might indicate whether a joint is undergoing persistent danger-DNA exposure. The use of EVs as liquid-biopsy carriers also has supporting evidence. Existing reviews indicate that the surface markers and cargo composition of EVs from patients with OA change with disease status, and some EV features are associated with disease severity and pain, supporting their potential use in disease stratification and biomarker development (Clarke et al., 2025). However, substantial differences remain in EV isolation methods, particle-size definitions, source-cell identification, and cargo quantification. No individual EV measure can yet serve as a mature companion diagnostic for cGAS-STING-targeted therapy.

Biomarkers should also reflect activity of the pathway itself rather than only the source of DNA. Candidate measures could include cGAS, STING, phosphorylated TBK1, and related interferon transcriptional signatures in tissues or cells, together with cGAMP levels to assess whether DNA sensing is actually occurring. Existing OA studies have identified cGAS-STING abnormalities in human cartilage and experimentally linked them to inflammation and matrix degeneration, but these assays have largely relied on tissue samples and have not yet yielded a standardized liquid-based testing system suitable for routine clinical use (Yang X. et al., 2024; Osburn et al., 2022). Selecting patients solely on the basis of increased STING staining in cartilage is therefore not yet practical in real-world clinical settings.

Third, inflammatory outputs can provide functional readouts. Studies of synovial fluid from human knee OA have identified distinct inflammatory biomarker profiles across patients. Soluble vascular cell adhesion molecule 1 (sVCAM-1), MMP-3, soluble intercellular adhesion molecule 1 (sICAM-1), tissue inhibitor of metalloproteinases 1 (TIMP-1), vascular endothelial growth factor (VEGF), and monocyte chemoattractant protein 1 (MCP-1) are associated to varying degrees with synovitis, imaging severity, or symptoms and correlate with markers of macrophage and neutrophil activation (Haraden et al., 2019). These markers are not specific to cGAS-STING, but they can help identify patients with a prominent inflammatory OA background. Their value may be greater when combined with mtDNA, EV features, and measures of STING-pathway activity than when any single marker is used alone.

A feasible precision-treatment model should not rely on a single STING biomarker but instead on a multilevel mechanistic combination. Patients with high levels of free or EV-associated mtDNA/mitochondrial DAMPs in synovial fluid, strong STING-TBK1 pathway activity, and pronounced inflammatory and catabolic outputs could provisionally be described as having a cGAS-STING-high OA endotype. “High activity” would not mean increased STING expression alone. It would require at least three mutually supporting mechanistic dimensions: first, increased upstream burdens of mtDNA and EV-associated danger signals; second, increased activity of DNA-sensing and signal-transduction steps such as cGAMP-STING-TBK1; and third, enhanced downstream interferon, NF-κB, SASP, inflammatory, or matrix-catabolic outputs. The value of this concept is that it directly links therapeutic mechanism to patient selection. If the dominant abnormality is mtDNA generation and leakage, mitochondrial protection and upstream danger-signal control may be prioritized. If STING-TBK1 activity remains high together with strong inflammatory output, local and stage-specific STING inhibition could be evaluated further. Importantly, however, all markers currently proposed for this endotype remain candidate biomarkers. Despite their mechanistic relevance, standardized sample-processing procedures, validated clinical assays, prespecified diagnostic thresholds, and replication in independent prospective cohorts are still lacking. These measures therefore cannot currently be used for routine clinical patient selection or treatment decisions. Establishing a cGAS-STING-high OA endotype will require at least three levels of validation. First, analytical methods must be standardized and their reproducibility assessed across centers. Second, prospective longitudinal cohorts must define the temporal relationships between these measures and disease progression, pain, synovitis, and structural change. Third, mechanism-targeted intervention studies must determine whether these biomarkers can predict treatment response (Figure 3).

## 6 Discussion and perspectives

Research on cGAS-STING is changing the conceptual framework used to explain inflammation in OA. Compared with the traditional view of inflammation as a phenomenon accompanying cartilage degeneration, current evidence supports a more dynamic model. Mechanical, metabolic, and aging-related injuries increase abnormal DNA exposure; DNA sensing then alters inflammation, cell fate, and matrix homeostasis; and the resulting new damage provides continued stimulation to innate immunity. Within this process, cGAS-STING is appropriately viewed as a potential pathological amplification node. A systematic review of cGAS-STING emphasized that the pathway contributes both to pathogen defense and to sensing endogenous DNA generated by cellular stress, and that its ultimate effects depend strongly on cell type and tissue context (Hooftman et al., 2026). In OA, however, the current evidence indicates that cGAS-STING-driven pathology mainly amplifies inflammation and tissue injury in particular experimental contexts or disease states with corresponding pathway activity. A more precise interpretation is therefore that cGAS-STING is an inflammatory amplification node whose causal weight varies across cell types, disease stages, and patient subgroups. Evidence for therapeutic benefit is derived predominantly from *in vitro* cell studies and experimental OA animal models. Human randomized clinical trials centered on cGAS- or STING-specific targeted drugs are still lacking. Several issues must therefore be resolved before this pathway can be used to prevent local injury from evolving into long-term whole-joint immune dysregulation.

The first issue is temporal causality. Improvement after drug treatment does not establish that STING in a particular cell type is an indispensable driver throughout the entire disease process, because an inhibitor may affect multiple joint compartments simultaneously. Future work should use temporally controlled conditional-knockout models in multiple intra-articular cell types to distinguish cell-autonomous effects from secondary tissue effects. This question also concerns where cGAS-STING lies within the course of OA. Longitudinal studies should measure DNA sources, cGAS-STING activity, and tissue phenotypes simultaneously at disease initiation, early inflammation, structural progression, and late remodeling. Such studies are needed to determine whether the pathway functions primarily as a disease initiator, a progression amplifier, or a pathway whose role changes across stages (Goldring and Goldring, 2016). Only after this temporal relationship is established can the therapeutic window be defined rationally.

The second issue is cell-type heterogeneity. The most complete direct evidence for cGAS-STING in OA currently comes from chondrocytes, yet OA is clearly not a disease of chondrocytes alone. Cross-tissue single-cell studies have identified active cellular communication among cartilage, synovium, meniscus, and subchondral bone. They have also reported fewer regenerative stromal cells, more osteogenic cells, and more macrophages with inflammatory and pain-associated features in OA joints (Raut RD. et al., 2025; Li J. et al., 2026). Whether these cells display cGAS-STING activity of the same magnitude and functional direction remains largely unknown. In particular, increased STING expression in whole tissue should not be taken to mean that every cell is in the same signaling state. Activation of cGAS-STING also involves cGAMP production, STING subcellular translocation, TBK1 recruitment, and downstream phosphorylation, so transcript abundance cannot fully represent actual pathway activity (Hooftman et al., 2026; Yan D. et al., 2026; Wang H. et al., 2026; Zhao K. et al., 2026). Single-cell transcriptomics therefore needs to be integrated with spatial technologies and protein-level measurements. OA pathology is highly regional: cell states differ markedly between intact and severely degenerated regions of the same cartilage specimen, and synovitis is often focal (Sato et al., 2006; Roemer et al., 2010). Advances in spatial transcriptomics now allow cellular states and signaling gradients in cartilage, synovium, subchondral bone, and periarticular tissues to be analyzed while preserving anatomical location (Xie et al., 2026). For cGAS-STING, an ideal future atlas should not merely map expression of the CGAS and STING1 gene symbols, which encode cGAS and STING, respectively. It should integrate spatial transcriptomics, cGAMP quantification, phosphorylated STING/TBK1 protein, cytosolic DNA localization, markers of mitochondrial injury, and histopathology to generate a spatial map that truly reflects pathway activity.

Another important translational issue is the immune safety window for cGAS-STING inhibition. cGAS-STING is a core component of cytosolic DNA recognition and host innate immune defense, and its physiological function is not limited to OA-associated sterile inflammation. Sustained and broad systemic inhibition could theoretically weaken innate immune responses to DNA pathogens and affect normal immune surveillance. For chronic OA, which may require years of management, long-term systemic shutdown of this pathway could therefore carry safety costs that differ from those observed in short-term animal experiments. These concerns support three strategies with stronger translational rationale. First, abnormal mtDNA generation and cytosolic leakage could be reduced preferentially, decreasing pathological input while preserving normal DNA sensing. Second, intra-articular local delivery could be used to limit systemic exposure. Third, inhibition could be intermittent or stage-specific according to pathway activity and disease stage rather than continuous and complete. Future preclinical studies should therefore evaluate not only chondroprotection but also infection susceptibility, systemic immune effects, local host defense, and pathway recovery after treatment withdrawal.

Research models also need to evolve. DMM and other surgically induced OA models are highly useful for analyzing rapid disease progression after mechanical injury, and a substantial proportion of key causal and pharmacological evidence in the cGAS-STING field has come from such models. They offer controlled timing, relatively reproducible lesions, and convenient genetic and pharmacological intervention. However, their rapid trauma-driven disease course cannot fully reproduce the pathology that develops gradually over years in most human OA. Human OA is typically influenced simultaneously by aging, obesity and metabolic abnormalities, previous injury, long-term mechanical loading, and genetic background. Pathway activation and drug protection observed in DMM models therefore cannot be equated directly with the causal importance of the same pathway in chronic human OA. Future studies should compare traumatic, aging-related, and metabolic OA models and increase the use of human cartilage explants, synovium-cartilage co-culture, multi-tissue joint-on-chip systems, and patient-derived models to test whether mechanistic chains established in animal experiments are reproducible and clinically relevant in human disease.

The final major issue is patient stratification, which requires reliable biomarkers. Early studies of synovial fluid demonstrated substantial inflammatory heterogeneity among patients with OA. Measures such as sVCAM-1, MMP-3, sICAM-1, TIMP-1, VEGF, and MCP-1 show varying associations with synovitis, imaging severity, or symptoms and correlate with markers of macrophage and neutrophil activation (Haraden et al., 2019; Xu et al., 2012; Okuyan et al., 2019; Bof et al., 2021; Daghestani and Kraus, 2015). Reviews of clinical-trial design for inflammatory OA endotypes suggest that imaging, inflammatory markers in synovial fluid, and tissue-remodeling markers may help identify patients more likely to benefit from anti-inflammatory treatment, but a unified and broadly validated tool for classifying individual patients is still lacking (Thudium et al., 2025). For cGAS-STING-directed treatment, general inflammatory markers alone are insufficient because they cannot distinguish inflammation driven by DNA sensing from other sources of innate immune activation. A more rational companion-biomarker system should cover multiple levels of the inflammatory amplification loop. Upstream measures could include free mtDNA, oxidized mtDNA, and EV-associated mtDNA in synovial fluid. Pathway-level measures could include cGAMP, activation states of STING and TBK1, and corresponding interferon-related transcriptional signatures. Downstream measures could include the SASP, chemokines, and matrix-degradation markers. Imaging could incorporate synovitis, bone-marrow lesions, and changes in cartilage structure. Such a combination does not imply that every measure must ultimately enter clinical practice. Longitudinal cohorts should first identify the smallest biomarker set that retains sufficient information and validate its relationship with symptoms, structural progression, and treatment response.

Another direction worth pursuing is to expand the therapeutic goal from suppressing inflammation to restoring the capacity for inflammatory resolution. The amplification loop persists not only because proinflammatory signaling is excessive, but also because damaged mitochondria, dead cells, aberrant DNA, and senescent cells are not cleared adequately. Lowering a single inflammatory mediator may transiently reduce tissue responses without eliminating the persistent source of danger signals. From this perspective, mitochondrial quality control, restriction of mtDNA, interruption of pathological EV propagation, and STING inhibition are not independent competing therapies. They may instead have complementary roles at different disease stages. OA research has already progressed from simply measuring STING expression to experimentally manipulating mechanosensing, mtDNA release, and intercellular DNA transfer. This progression provides an experimental basis for shifting therapy from downstream anti-inflammatory suppression toward danger-signal control and restoration of homeostasis.

Overall, cGAS-STING provides an integrative mechanistic framework linking mechanical injury, mitochondrial dysfunction, aberrant DNA exposure, inflammation, and tissue degeneration in OA. As an amplification node that is tightly coupled to other pattern-recognition systems, inflammasomes, metabolic stress, and intercellular communication, its contribution varies with cell type, patient background, and disease stage. The key transition now required in this field is to move from documenting pathway abnormalities to defining therapeutically actionable disease states. Temporally controlled and cell-specific genetic studies should first determine the causal contribution of cGAS-STING at different stages of OA. Single-cell and spatial multi-omics should then identify where abnormal signals originate, which cells they affect, and how they propagate across tissues, while research models are upgraded to better reflect contemporary human OA populations. At the level of patient stratification, individuals and time windows with a greater cGAS-STING contribution should be identified along the continuous biological spectrum of OA. Ultimately, the goal of breaking the OA inflammatory amplification loop is not to permanently disable innate immunity but to restore balance among damage generation, danger sensing, inflammatory output, and tissue repair. If abnormal signal sources can be reduced promptly in patients with persistent immunogenic DNA generation, if local and stage-specific inhibition can be applied when pathological STING activity is high, and if organelle quality control and repair capacity can be restored after tissue remodeling has occurred, cGAS-STING could develop from a molecular axis that explains OA inflammation into a translational hub linking patient stratification, biomarkers, and disease-modifying therapy. The critical task is not to show that more inflammatory molecules are associated with STING, but to determine when, where, and in which patients this amplification network should be interrupted to prevent local injury from progressing to long-term whole-joint immune dysregulation.

## 7 Conclusion

In summary, the cGAS-STING pathway provides an integrative mechanistic framework for understanding the transition of osteoarthritis from local tissue injury to chronic whole-joint inflammation. Abnormal mechanical loading, aging, oxidative stress, and metabolic stress in OA first disrupt nuclear and mitochondrial homeostasis. Among these processes, mitochondrial dysfunction and cytosolic mtDNA leakage currently constitute the best-supported endogenous source of DNA danger signals. Once recognized through cGAS-STING, aberrant DNA can promote inflammation and matrix catabolism through pathways including IRF3/IFN-I and NF-κB and can interact with inflammasomes, pyroptosis, and cellular senescence. The resulting inflammation further induces organelle injury, cell death, and ECM degradation, continuously replenishing sources of DNA and DAMPs and gradually converting a potentially reversible injury response into a self-reinforcing inflammatory amplification loop. Importantly, this process is not confined to individual chondrocytes. Cytokines, the SASP, chemotactic signals, and EVs carrying miRNAs, proteins, and mtDNA establish bidirectional communication among cartilage, synovium, the infrapatellar fat pad, and subchondral bone, allowing inflammation to expand from local cellular responses into whole-joint tissue remodeling. cGAS-STING is therefore better defined as a key amplification node within the OA pathological network than as a single shared initiating cause in all patients with OA. At present, treatment of this network should gradually shift from simple downstream anti-inflammatory therapy toward control of danger signals and restoration of homeostasis. Early intervention may focus on reducing mechanical and mitochondrial injury and mtDNA leakage. During persistent inflammation, local and moderate cGAS-STING inhibition may be appropriate. Once pyroptosis, the SASP, synovial fibrosis, and osteochondral remodeling are established, combined blockade of downstream feedback together with promotion of tissue repair may be required. The central task for future research is not to demonstrate additional associations between inflammatory molecules and STING, but to define the causal contribution of this pathway at different disease stages through cell-specific and temporally controlled studies. Spatial multi-omics, synovial-fluid mtDNA, cGAMP, and measures of STING/TBK1 activity should be integrated into a quantifiable pathway-activity assessment system to identify patients with genuinely high cGAS-STING activity and the corresponding therapeutic windows. The ultimate therapeutic goal is not permanent shutdown of innate immunity, but restoration of balance among damage generation, danger sensing, inflammatory resolution, and tissue repair, thereby preventing OA from progressing from local injury to persistent whole-joint immune dysregulation.
